# Risk Factors for Major Adverse Cardiovascular Events, Malignancies, and Serious Infections With Tofacitinib in Rheumatoid Arthritis: Post Hoc Analysis of a 3‐Year J‐Post‐Marketing Surveillance

**DOI:** 10.1111/1756-185x.70572

**Published:** 2026-02-09

**Authors:** Kunihiro Yamaoka, Masato Hoshi, Yutaka Endo, Toshitaka Hirano

**Affiliations:** ^1^ Department of Rheumatology Kitasato University School of Medicine Sagamihara Kanagawa Japan; ^2^ Pfizer Japan Inc. Tokyo Japan; ^3^ Pfizer R&D Japan G.K Tokyo Japan

**Keywords:** Janus kinase inhibitor, major adverse cardiovascular events, malignancy, post hoc analysis, rheumatoid arthritis, serious infection, tofacitinib

## Abstract

**Aim:**

This post hoc analysis of all‐case post‐marketing surveillance in Japanese patients with rheumatoid arthritis assessed risk factors for major adverse cardiovascular events (MACE), malignancies, and serious infections (SI).

**Methods:**

Data of patients who received tofacitinib (≥ 1 dose) were stratified by each combination of age (< 50 years and ≥ 50 years) and the number of cardiovascular (CV) risk factors (0 or ≥ 1). Incidence rates (IRs) of each adverse event (AE)‐MACE, malignancies, and SI, stratified by age, CV risk factors, and patient background factors were calculated.

**Results:**

Overall, 7021 patients were stratified by each combination of age (< 50 and ≥ 50 years) and the number of CV risk factors (0 or ≥ 1). The IRs for malignancies and SI were higher in patients aged ≥ 50 vs. < 50 years. The IRs for all AEs were higher in patients with ≥ 1 CV risk factors than 0 CV risk factors. Older age was strongly correlated with all AEs. MACE and SI were more likely to be affected by any CV risk factors, while the other AE was influenced by some of them.

**Conclusions:**

Older age was a risk factor for all AEs (MACE, malignancies, and SI). MACE risk was also increased in patients with ≥ 1 CV risk factors versus those without. SI risk might also be increased in patients with ≥ 1 CV risk factors.

## 1 Introduction

Rheumatoid arthritis (RA) is a chronic, immune‐mediated inflammatory disease, which leads to disability and increases mortality [1, 2]. In Japan, a nationwide population‐based questionnaire survey reported an estimated RA prevalence of 0.75% and demonstrated its adverse impact on patients' quality of life [3]. Tofacitinib, a Janus kinase inhibitor (JAKi), was approved in Japan for the treatment of rheumatoid arthritis (RA) in patients with an inadequate response to ≥ 1 disease‐modifying antirheumatic drugs [4]. Clinical evidence [5, 6, 7, 8, 9, 10, 11, 12, 13, 14, 15, 16, 17], including long‐term extension studies [18, 19, 20], has demonstrated the safety and efficacy of tofacitinib (5 mg and 10 mg twice daily) either as monotherapy or in combination with methotrexate in patients with RA.

In patients with RA, a high risk of adverse events (AEs) such as infections [21], malignancies [22], cardiovascular (CV) events and venous thromboembolism (VTE) [23] has been reported, which further increase with immunosuppressive treatments such as JAKi [24]. The Oral Rheumatoid Arthritis trial (ORAL) Surveillance study evaluated patients with active RA (despite methotrexate treatment) who received tofacitinib (5 mg or 10 mg twice daily) or a subcutaneous tumor necrosis factor inhibitor (TNFi) and were ≥ 50 years of age and had ≥ 1 CV risk factors. Despite comparable efficacy, tofacitinib was associated with a higher risk of major adverse cardiovascular events (MACE) and malignancies than TNFi. The study did not meet the prespecified non‐inferiority criteria for MACE and malignancies in the primary comparison of tofacitinib vs. TNFi [25]. Furthermore, post hoc analyses of the ORAL Surveillance showed that major risk factors contributing to the excess risk observed with tofacitinib vs. TNFi for MACE and malignancies were age ≥ 65 years, current or past long‐term smoking, and atherosclerotic CV disease (specifically for MACE), and atherosclerotic CV disease [22, 23, 26]. However, the ORAL Surveillance primarily included populations from Western countries (59.4%) and other regions (40.6%), with 4.0% from Asia excluding Japan [25].

The all‐case post‐marketing surveillance (PMS) study evaluated real‐world safety and effectiveness of tofacitinib in Japanese patients with RA [24]. It was hypothesized that the safety outcomes, particularly the incidences of MACE and malignancies, might differ by geography, considering differences in the prevalence of cancer subtypes [27]. These results were consistent with the known safety profile of tofacitinib [24]. Moreover, results from the 3‐year final analysis of the PMS showed that the incidence rates (IRs) of SI were higher, and the IRs of malignancies were numerically higher with tofacitinib than with control (other drugs). However, these results should be interpreted cautiously due to unmeasured confounders [28].

We believe that the PMS data are of great interest for providing valuable insights into risk factors associated with safety outcomes of tofacitinib in patients with RA. Therefore, we aimed to conduct a post hoc analysis using the PMS data to confirm the IRs of MACE and malignancies, stratified by patients' age and CV risk factors as identified in the ORAL Surveillance study [25]. The analysis also aimed to identify risk factors associated with AEs of MACE, malignancies, and SI in the PMS population.

## 2 Materials and Methods

### 2.1 Study Design and Patients

We conducted a post hoc analysis using data from a 36‐month PMS study of tofacitinib in patients with RA in Japan (NCT01932372; from 30 July 2013 to 24 August 2021) [24]. Japanese patients with RA who received tofacitinib, regardless of methotrexate use, were enrolled in this PMS study [24]. Data were collected from all sites where tofacitinib was prescribed. The safety analysis set included all patients who received ≥ 1 dose of tofacitinib.

In this post hoc analysis, data of patients were stratified by each combination of age (< 50 years and ≥ 50 years) and the number of CV risk factors (0 or ≥ 1). The CV risk factors that were defined in the ORAL Surveillance study were used: current cigarette smoking, hypertension, high‐density lipoprotein (HDL) cholesterol level < 40 mg/dL, diabetes mellitus, family history of premature coronary heart disease, extra‐articular RA, and/or history of coronary artery disease (CAD). Family history of premature coronary heart disease was not collected in the PMS study and therefore could not be included as a CV risk factor. Additionally, patients with missing information on a CV risk factor were considered as absence of a CV risk factor. Considering the impact of older age on these risk factors, further stratification was performed for patients with RA aged ≥ 50 years (< 50 years, ≥ 50 to < 65 years, and ≥ 65 years). Additionally, the IR of each AE by CV risk factors and other patient background factors were evaluated. All patient groups were assessed for the incidence of AEs and associated risk factors over 3 years from the start of treatment.

### 2.2 Study Ethics

The PMS study was conducted in compliance with the “Good Post‐marketing Study Practice” (GPSP Ordinance; Ministry of Health, Labour and Welfare Ordinance No. 171, dated 20 December 2004) of Japan. In accordance with GPSP, approval by institutional review boards was not required. As patients received tofacitinib as part of their routine medical care, informed consent was not required unless mandated by the medical institution. Written contracts were signed by all medical institutions before the provision of tofacitinib for prescription to patients.

### 2.3 Outcomes

The proportions of patients with all‐causality and treatment‐related AEs and serious AEs were assessed (coded using the English–Japanese Medical Dictionary for Regulatory Activities [MedDRA/J], version 23.1).

In this post hoc analysis, IRs for the following AEs of special interest were evaluated as important risks with tofacitinib: MACE (defined as investigator‐reported death from CV causes, nonfatal myocardial infarction, or nonfatal stroke), malignancies, and SI, as defined in Table S1. The IRs were evaluated using data stratified by each combination of age (< 50 years and ≥ 50 years) and the number of CV risk factors (0 or ≥ 1). The IRs were also assessed by CV risk factors and other patient background factors.

Patient background factors included sex, weight, duration of disease, stages of RA, class of RA, history of various diseases, complications, status of methotrexate use, status of oral steroid use, initial tofacitinib daily dose, and history of tacrolimus and biological agent use.

### 2.4 Statistical Analysis

Patient background factors were reported descriptively, stratified by each combination of age (< 50 years, ≥ 50 to < 65 years, and ≥ 65 years) and the number of CV risk factors (0 or ≥ 1).

IRs, with 95% confidence intervals (CIs) based on the Poisson distribution, were calculated for the AEs. IRs were expressed as number of events per 100 patient‐years (PY). Duration of exposure (PY) was calculated from the treatment start date to the onset date of each AE or the censoring date (in days) divided by 365.25. If no events were observed, censoring occurred at the treatment completion or discontinuation date, except for malignancies. Malignancies were counted within a predefined risk period, defined as the treatment start date to the onset date of malignancies or the last follow‐up date (i.e., the latest of event start date, event stop date, last visit date, withdrawal date, telephone contact date, or death date). Patients without malignancy events were censored at the last follow‐up date.

The incidence proportion and rate of each AE were analyzed by each combination of age (< 50 years, ≥ 50 to < 65 years, and ≥ 65 years) and the number of CV risk factors (0 or ≥ 1), and patient background factors as candidates of risk factors. Cox proportional hazards model was used to identify risk factors associated with each AE, performed on all patients with “unknown” included as a category, and hazard ratios (HRs) with 95% CIs were calculated.

Univariable analyses were performed to test each factor independently, with *p* < 0.10 as the threshold for inclusion of the covariates in the multivariable analyses. The selected factors were then tested in stepwise multivariable analyses, performed on all patients, with *p* < 0.05 set as the level of statistical significance. As a sensitivity analysis, a multiple imputation method was performed. Missing data of covariates were imputed by random forests using a fully conditional specification (FCS) algorithm. After the imputation, the same analyses were performed for each created data set. The results obtained from each data set were summarized based on Rubin's rule [29]. As another sensitivity analysis, the exposure period for malignancies was defined as the on‐treatment period plus 90 days, because the initially defined exposure period might be too long to regard the events as being caused by the exposure. Statistical software is Windows SAS 9.4 TS Level 1M8.

## 3 Results

### 3.1 Patient Background Factors by Each Combination of Age and the Number of CV Risk Factors

Of the 7021 patients, 1901 were aged ≥ 65 years without any CV risk factors, followed by those aged ≥ 65 years with ≥ 1 CV risk factors (*n* = 1724), those aged ≥ 50 to < 65 years without CV risk factors (*n* = 1441), those aged ≥ 50 to < 65 years with ≥ 1 CV risk factors (*n* = 843), those aged < 50 years without CV risk factors (*n* = 844), and those aged < 50 years with ≥ 1 CV risk factors (*n* = 268). RA stage I–II was common in patients aged < 50 years (both with and without CV risk factors). The majority of patients in all groups had RA class 1–2 and no extra‐articular disease associated with RA. There was a trend toward increasing numbers of patients with stage IV, class 4 disease, or RA duration ≥ 20 years with advancing age. Among patients with ≥ 1 CV risk factors, smoking history was most common in those aged < 50 years, followed by those aged ≥ 50 to < 65 years and ≥ 65 years, whereas hypertension and diabetes were more frequent in patients aged ≥ 65 years, followed by those aged ≥ 50 to < 65 years and < 50 years. Most patients across all groups had no history of CAD, infection, herpes zoster, CV disease, malignancies, lung disorder, interstitial pneumonia, metabolic abnormality, tacrolimus use, or family history of malignancies. Moreover, the majority of patients did not experience complications such as malignancies, infection, herpes zoster, CV disease, or lung disease (Table S2).

### 3.2 IRs of AEs Stratified by Each Combination of Age and the Number of CV Risk Factors

**FIGURE 1:**
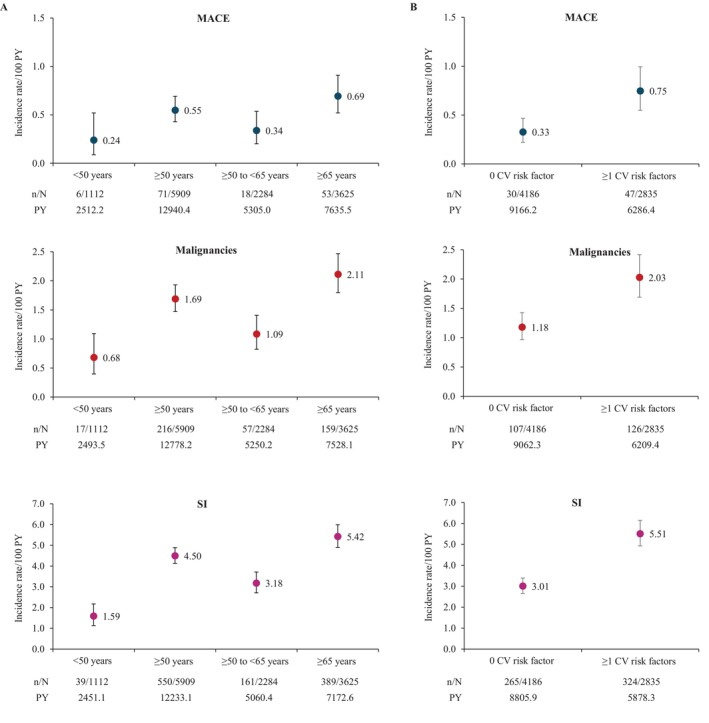
IRs of AEs stratified by (A) age and (B) the number of CV risk factors. *n* indicates the number of patients with the event. *N* indicates the total number. Error bars represent the 95% CIs. AE, adverse event; CI, confidence interval; CV, cardiovascular; IR, incidence rate; MACE, major adverse cardiovascular events; PY, patient‐years; RA, rheumatoid arthritis; SI, serious infection.

**FIGURE 2:**
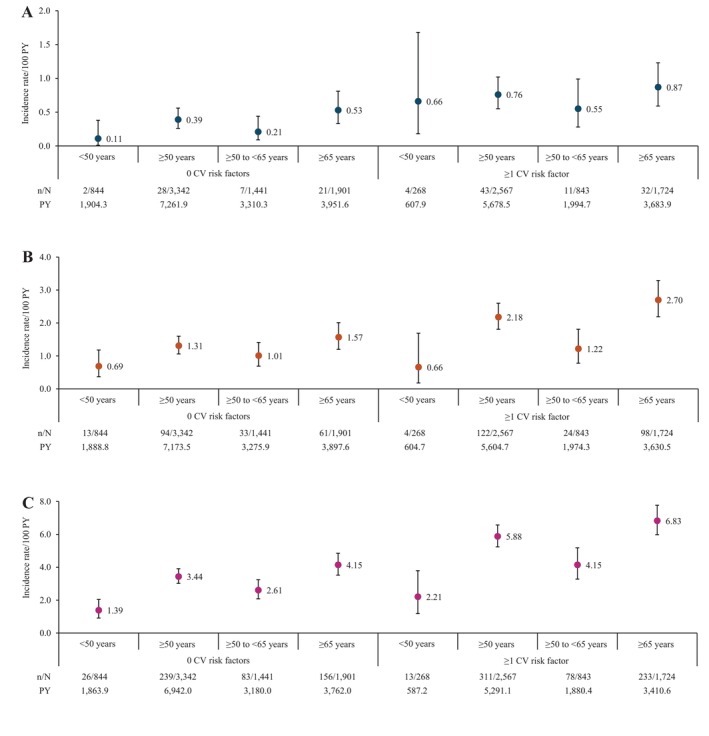
IRs of AEs: (A) MACE, (B) malignancies, and (C) SI, stratified by each combination of age and the number of CV risk factors. *n* indicates the number of patients with the event. *N* indicates the total number of patients. Error bars represent the 95% CIs. AE, adverse event; CI, confidence interval; CV, cardiovascular; IR, incidence rate; MACE, major adverse cardiovascular events; PY, patient‐years; RA, rheumatoid arthritis; SI, serious infection.

The IRs for malignancies and SI were higher in patients aged ≥ 50 years vs. < 50 years. The IRs for MACE, malignancies and SI were higher in patients with ≥ 1 CV risk factors than in those without CV risk factors (Figure 1). The IR (95% CI) for MACE was 0.76 (0.55–1.02), 0.39 (0.26–0.56), 0.66 (0.18–1.68), and 0.11 (0.01–0.38) for each combination of age (≥ 50 years vs. < 50 years) and the number of CV risk factors (≥ 1 vs. 0), respectively (Figure 2A). The IR (95% CI) for malignancies was 2.18 (1.81–2.60), 1.31 (1.06–1.60), 0.66 (0.18–1.69), and 0.69 (0.37–1.18), respectively (Figure 2B). The IR (95% CI) for SI was 5.88 (5.24–6.57), 3.44 (3.02–3.91), 2.21 (1.18–3.79), and 1.39 (0.91–2.04), respectively (Figure 2C).

### 3.3 MACE

The HR of MACE was the highest in patients aged ≥ 50 years with ≥ 1 CV risk factors, with an HR of 7.16, followed by 6.25 in those aged < 50 years with ≥ 1 CV risk factors, and 3.68 in those aged ≥ 50 years without CV risk factors, compared with patients aged < 50 years without CV risk factors (Table 1). Patients aged ≥ 50 years and ≥ 1 CV risk factors had higher risk. As a result after performing multiple imputation, HR were 9.77, 8.21, and 5.10, respectively (Table S6.1). Although there was no qualitative change, there was a quantitative change, that means the risk might increase in patients aged ≥ 50 years and ≥ 1 CV risk factors.

**TABLE 1:** Risk of AEs stratified by each combination of age and the number of CV risk factors.

AEs		< 50 years		≥ 50 years		≥ 50 to < 65 years		≥ 65 years	
		0 CV risk factor (*n* = 844)	≥ 1 CV risk factors (*n* = 268)	0 CV risk factor (*n* = 3342)	≥ 1 CV risk factors (*n* = 2567)	0 CV risk factor (*n* = 1441)	≥ 1 CV risk factors (*n* = 843)	0 CV risk factor (*n* = 1901)	≥ 1 CV risk factors (*n* = 1724)
MACE	HR (95% CI)	Referent	6.25 (1.14–34.12)	3.68 (0.88–15.44)	7.16 (1.74–29.56)	2.01 (0.42–9.68)	5.13 (1.14–23.15)	5.10 (1.20–21.76)	8.25 (1.98–34.42)
	*p*	—	0.034	0.075	0.006	0.384	0.033	0.028	0.004
Malignancies	HR (95% CI)	Referent	0.96 (0.31–2.94)	1.91 (1.07–3.41)	3.15 (1.78–5.58)	1.47 (0.77–2.79)	1.75 (0.89–3.44)	2.26 (1.24–4.11)	3.94 (2.21–7.02)
	*p*	—	0.940	0.029	< 0.001	0.241	0.103	0.008	< 0.001
SI	HR (95% CI)	Referent	1.58 (0.81–3.07)	2.42 (1.62–3.63)	4.12 (2.76–6.15)	1.87 (1.20–2.91)	2.97 (1.90–4.63)	2.87 (1.89–4.34)	4.76 (3.17–7.13)
	*p*	—	0.181	< 0.001	< 0.001	0.005	< 0.001	< 0.001	< 0.001

*Note:* *p* < 0.05 indicates statistical significance. HRs were adjusted for covariates (age and CV risk factors) and estimated using a Cox proportional hazards model. Number of patients with the event and patient‐years are shown in Figure 2.

Abbreviations: AE, adverse event; CI, confidence interval; CV, cardiovascular; HZ, herpes zoster; MACE, major adverse cardiovascular events; ND, not detected; SI, serious infection.

**FIGURE 3:**
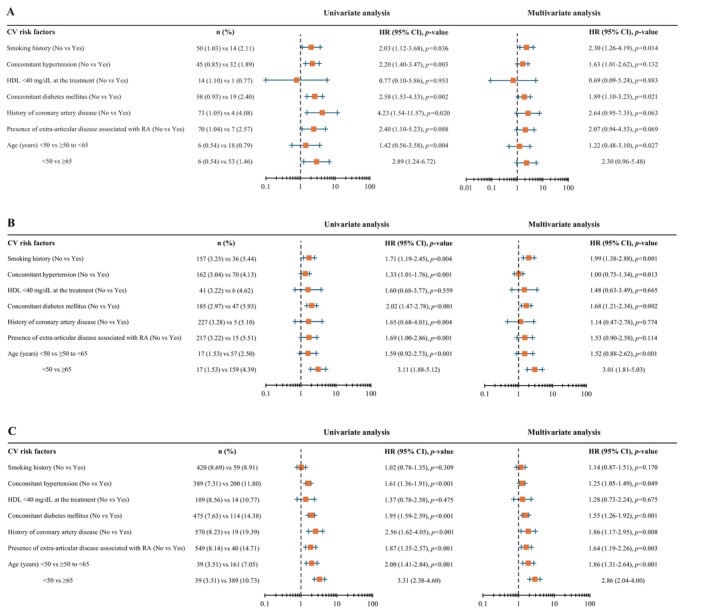
CV risk factors for the incidence of AEs: (A) MACE, (B) malignancies, and (C) SI. In multivariable analysis, CV risk factors with *p* < 0.05 indicate statistical significance. The reference category for all CV risk factors was “No”, except for age, where the reference category was “< 50 years”. AE, adverse event; CI, confidence interval; CV, cardiovascular; HDL, high‐density lipoprotein; HR, hazard ratio; MACE, major adverse cardiovascular events; RA, rheumatoid arthritis; SI, serious infection.

Among the CV risk factors, smoking history, concomitant diabetes mellitus, and older age were statistically significant risk factors for MACE in multivariable analyses. Although not statistically significant, HRs were slightly higher for other factors such as concomitant hypertension, history of CAD, and presence of extra‐articular disease associated with RA. That means almost all the CV risk factors except for HDL had the possible risk (Figure 3). HRs after performing multiple imputation were similar (Table S7.1).

In multivariate analyses, patient background factors found as statistically significant for MACE were as follows: smoking history, stages of RA, class of RA, history of CV disease, complication of CV disease, and status of methotrexate use (Table S3). As a result after performing multiple imputation, concomitant diabetes mellitus, status of oral steroid use, and history of tacrolimus use were statistically significant, and stages of RA and class of RA were not (Table S8.1).

### 3.4 Malignancies

The HR of malignancies was the highest in patients aged ≥ 50 years with ≥ 1 CV risk factors, with an HR of 3.15, followed by 1.91 in those aged ≥ 50 years without CV risk factors, and 0.96 in those aged < 50 years with ≥ 1 CV risk factors, compared with patients aged < 50 years without CV risk factors (Table 1). HRs after performing multiple imputation were similar (Table S6.2).

In multivariable analyses, smoking history, concomitant diabetes mellitus, and older age were statistically significant CV risk factors for malignancies. Concomitant hypertension was not identified as a risk factor, as the HR with patients with it was 1.00 compared with those without it, although it was statistically significant. Moreover, the HRs for history of CAD and presence of extra‐articular disease associated with RA were slightly higher but not statistically significant (Figure 3). As the numbers were extremely small, it was difficult to evaluate whether the presence or absence of CV risk factors in patients was associated with risk of malignancies. The result after performing multiple imputation was similar. It might become clear that concomitant hypertension was not a risk factor (Table S7.2).

Patient background factors identified as statistically significant for malignancies were as follows: smoking history, concomitant diabetes mellitus, male sex, older age, higher body weight, history of infection, history of metabolic abnormality, complication of malignancies, and history of biological agent use (Table S4). The result after performing multiple imputation was similar. It should be noted that history of metabolic abnormality was not statistically significant (Table S8.2).

All the results after changing the definition of exposure period as the on‐treatment period plus 90 days were similar (Tables S9.1–S9.3).

### 3.5 SI

Compared with patients aged < 50 years without CV risk factors, the risk of SI was the highest with an HR of 4.12 in patients aged ≥ 50 years with ≥ 1 CV risk factors, followed by 2.42 in those aged ≥ 50 years without CV risk factors, and 1.58 in those aged < 50 years with ≥ 1 CV risk factors (Table 1). HRs after performing multiple imputation were similar (Table S6.3).

In multivariable analyses, concomitant hypertension, concomitant diabetes mellitus, history of CAD, presence of extra‐articular disease associated with RA, and older age were statistically significant CV risk factors for SI (Figure 3). The result after performing multiple imputation was similar (Table S7.3).

Patient background factors identified as statistically significant were as follows: concomitant diabetes mellitus, history of CAD, older age, lower body weight, class of RA, duration of disease, history of infection, history of lung disorder, complication of malignancies, complication of lung disorder, complication of interstitial pneumonia, and status of oral steroid use. Multivariable analyses of patient background factors for SI are shown in Table S5. As a result after performing multiple imputation, history of herpes zoster, complication of cardiovascular disease, and status of MTX use were detected statistically significant. Class of RA and complication of interstitial pneumonia were not statistically significant (Table S8.3).

## 4 Discussion

This post hoc analysis was conducted to analyze the IRs for each AE (MACE, malignancies, and SI) in patients with RA stratified by each combination of age and the number of CV risk factors in the cohort of uncontrolled, single‐arm PMS conditioned based on the criteria of ORAL surveillance study, to add another line of detailed insights of the characteristics of tofacitinib AE prescribed for Japanese patients.

In the findings of this post hoc analysis, patient background factors varied between those aged < 50 years and those aged ≥ 50 years. Patients aged ≥ 50 years more often had ≥ 1 CV risk factors. Smoking history was mostly observed in patients aged < 50 years, whereas older patients more commonly had comorbid hypertension and diabetes. Regardless of age, the proportion of patients with RA with a history of CAD and extra‐articular disease associated with RA was very low. The IRs of all the AEs were higher with age. The IRs of MACE and SI were higher in patients with ≥ 1 CV risk factors.

Our results showed the IRs for all AEs were higher in patients aged ≥ 50 years with ≥ 1 CV risk factors than those reported in the PMS population [28]. In a post hoc analysis of the ORAL Surveillance study aimed at identifying subpopulations with different risks with tofacitinib vs. TNFi, the IRs for malignancies (3.05) were the highest, followed by MACE (2.37) in patients aged ≥ 65 years and ever smoked [23]. The study showed that patients aged ≥ 65 years or current/past smokers had a higher risk of malignancies (excluding nonmelanoma skin cancer; HR: 2.42 vs. 1.24) and MACE (2.74 vs. 1.09) with tofacitinib (vs. TNFi) [23]. Other evidence has also reported high IRs of MACE and malignancies with tofacitinib in older patients (≥ 65 years) with atherosclerotic CV disease [22, 23, 26]. The IR (95% CI) of VTE was reported 0.17 (0.12–0.25) in tofacitinib PMS [28].

In this study, the HR for risk of MACE showed that both age and the presence of CV risk factors affected the incidence of MACE. Among CV risk factors, older age (HR: < 50 years vs. ≥ 50 to < 65 years: 1.22; < 50 years vs. ≥ 65 years: 2.30), smoking history (2.30), and concomitant diabetes mellitus (1.89) were identified as risk factors associated with MACE. A prior post hoc analysis of the ORAL Surveillance study also reported that the subpopulation aged ≥ 65 years with long‐term smoking history was at higher risk of MACE [23]. In our findings, numerical associations (not statistically significant) with risk of MACE were also observed in patients with concomitant hypertension (HR: 1.63), history of CAD (2.64), and presence of extra‐articular disease associated with RA (2.07). However, these associations should be interpreted with caution due to the small sample sizes (CAD, *n* = 98; extra‐articular disease, *n* = 272), which reduced statistical precision. Furthermore, HDL was difficult to evaluate as a risk factor for MACE due to the extremely high number of unknowns (*n* = 5617). As a sensitivity analysis, multiple imputation was performed to impute missing data. HRs after performing this method were similar to the result we got initially. That means almost all the CV risk factors could be the possible risks and HDL was not identified as a risk factor. Among patients' background characteristics, a history or complication of CV disease (HR: 3.24 and 2.26, respectively) was found to be associated with the risk of MACE, which is consistent with previous reports indicating a higher risk of MACE with tofacitinib (vs. TNFi) in patients with RA with a history of atherosclerotic CV disease [26]. Moreover, among patients with CV disease complications (*n* = 1917), the majority (*n* = 1695) had hypertension. Additionally, CV disease includes illness of high severity (such as angina pectoris, myocardial infarction, cardiac failure, and arrhythmia); the high prevalence of hypertension may have masked the risk of more severe cardiovascular events. Although it was not clear how the history of CV disease was temporally related to MACE, the proportion of patients with a history of CV disease tended to be higher in older age groups in the patient background and clinical characteristics. This may suggest that age‐related CV disease complications are a risk factor for MACE. The risk of MACE also appeared related to the stage (HR: Stages I–II vs. IV: 1.39) and class (classes 1–2 vs. 3: 1.71; classes 1–2 vs. 4: 2.32) of RA. However, the HR was higher (44.68) in patients with an unknown stage of RA, indicating that caution should be applied when RA stage would be interpreted as a risk factor for MACE. The risk of MACE onset tended to be higher with increasing disease activity, which is consistent with earlier reports showing a similar trend between the risk of MACE with persistent inflammation and elevated disease activity [25, 30]. The risk of MACE was higher in patients not using methotrexate (HR: ≤ 8 mg/week: 0.66; > 8 mg/kg: 0.38) at the start of tofacitinib treatment. As the proportion of patients not using methotrexate was higher in the older age group, this association might partly reflect age‐related factors. The results after performing multiple imputation differed; concomitant diabetes mellitus, status of oral steroid use, and history of tacrolimus use were statistically significant, and stages of RA and class of RA were not. However, there may not be any new findings because it is already concerned that they may have the possible risk.

The findings of malignancy risk suggest that older age is a risk factor, but it is difficult to conclude whether the presence of CV risk factors independently increases malignancy risk. A previous study also showed an increased risk of malignancies with tofacitinib in patients with RA aged ≥ 50 years with CV risk factors [22]. Among CV risk factors in patients, smoking history (HR: 1.99), concomitant diabetes mellitus (1.68), and age (< 50 years vs. ≥ 50 to < 65 years: 1.52; < 50 years vs. ≥ 65 years: 3.01) were identified as statistically significant risk factors for malignancies, aligning with earlier reports [22, 23, 25]. Concomitant hypertension should not be considered a definitive risk factor, as the HR was high at 19.58 for unknown cases and 1.00 for those with hypertension. Furthermore, as mentioned previously in the MACE analysis, the history of CAD (1.14) and the presence of extra‐articular disease associated with RA (1.53) were difficult to evaluate as malignancy risk factors due to extremely small numbers. HRs after performing this method were similar and not affecting the main results. Concomitant hypertension and history of CAD were not identified as risk factors and the presence of extra‐articular disease associated with RA was a possible risk factor. Male sex was also linked to malignancy risk, as confirmed in a previous report [27]. The results also showed that higher weight (HR: < 50 kg vs. ≥ 50 to < 60 kg: 1.83; < 50 kg vs. ≥ 60 to < 70 kg: 1.35; < 50 kg vs. ≥ 70 kg: 1.25) tended to increase the risk of malignancies. Although weight alone may not be necessarily directly indicated the degree of obesity, this result may be showing that body type could be potential risk for malignancy in Japanese RA patients. A higher malignancy risk was observed in patients with a history of infection (HR = 1.50), which could likely be related to aging, as the proportion of patients with past infections was high among older patients, as observed in patient demographics. The history of metabolic abnormality might have been statistically significant due to the extremely high HR in the unknown category (673.21) or the slightly high HR in those with metabolic abnormality. As noted with concomitant diabetes mellitus and weight, the risk associated with abnormal lipids cannot be ruled out. Moreover, the number of patients meeting the criteria was extremely small (*n* = 96), and the lower limit of the 95% CI for the HR was below 1; therefore, it remains uncertain how much history of metabolic abnormality would actually affect malignancies over time. Regarding the history of biological agent use, due to the lack of details on the number and duration of use, its influence on malignancy risk remains unclear. The study also included 40 patients with preexisting malignancies at the start of tofacitinib treatment; thus, it is possible that progression or metastasis (HR: 28.23) could account for the appearance of malignancies as a risk factor. HRs after performing multiple imputation were similar. History of metabolic abnormality was not identified as a risk factor. It should be noted that the initial definition of the exposure period did not affect the conclusion, since all the results after changing the definition of exposure period were similar.

The findings of our study showed that both older age and the presence of CV risk factors contribute to the risk of SI. The statistically significant CV risk factors were concomitant hypertension (HR: 1.25), concomitant diabetes mellitus (1.55), history of CAD (1.86), presence of extra‐articular disease associated with RA (1.64), and age (< 50 years vs. ≥ 50 to < 65 years: 1.86; < 50 years vs. ≥ 65 years: 2.86). However, the numbers were very small, and statistical precision was low for patients with a history of CAD and the presence of extra‐articular disease. Nonetheless, considering the history of CAD as part of CV disease risk, it could be a possible risk factor for SI. Additionally, extra‐articular disease associated with RA was not statistically significant in patients' background and clinical characteristics; therefore, it was not clear whether it was a risk factor for SI. Although HRs after performing multiple imputation were similar, statistical precision was still low for patients with a history of CAD and the presence of extra‐articular disease. These findings suggested that age might increase the risk of SI. Similar findings were also observed in a previous report [21], which identified age as a risk factor for SI. Moreover, lower body weight (HR: < 50 kg vs. ≥ 50 to < 60 kg: 0.72; < 50 kg vs. ≥ 60 to < 70 kg: 0.83), RA class (classes 1–2 vs. 3: 1.21; classes 1–2 vs. 4: 1.20), and disease duration (< 2 years vs. ≥ 2 to < 5 years: 1.03; < 2 years vs. ≥ 5 to < 10 years: 1.31; < 2 years vs. ≥ 10 to < 20 years: 1.54; < 2 years vs. ≥ 20 years: 1.61) were also found to affect the risk of SI. In contrast to the higher weight associated with MACE and malignancies, lower weight was identified as a risk factor for SI. Aligning with our findings, an increased risk of SI with each 5 mg/L increment in serum C‐reactive protein levels and with higher disease activity was also reported previously [30]. Although malignancy related complications were associated with SI, a direct causal relationship remains unclear, as factors such as age or RA disease activity may be involved in malignancies. Furthermore, although the degree of susceptibility to infection in patients with a history of infection is unknown, a history of infection could increase the risk of SI (HR: 1.72). Similarly, a history of lung disorder was found to increase the risk of SI (HR: 1.51), which aligns with previous reports [21]. Moreover, an increased risk with steroid use (vs. no steroid use), with slight dose dependence (HR: < 2.5 mg/day: 0.95; ≥ 2.5 to < 5 mg/day: 1.20; ≥ 5 mg/day: 2.07) was observed in our study, showing a tendency similar to that reported previously [21]. Although HR after performing multiple imputation, history of herpes zoster, complication of cardiovascular disease and status of MTX use were newly statistically significant and class of RA and complication of interstitial pneumonia was not statistically significant, it was considered that this result did not change the conclusion regarding the possibility that certain medical histories, prior treatments or disease conditions might be the risk factors.

This study has several strengths. This post hoc analysis was conducted using PMS data, which included all patients with RA who received tofacitinib in Japan. Additionally, this analysis considered patients who were < 50 years old and without CV risk factors as the reference group, providing a better comparator for older age groups, a population that was not represented in the ORAL Surveillance study. Some limitations of this additional analysis should also be considered when interpreting the findings. The additional analysis was post hoc and exploratory. The PMS study did not include a control group; hence, the risk attributable to tofacitinib itself could not be evaluated. Since these are additional analyses, some of the HRs and statistical test results were difficult to interpret in terms of whether the statistically selected factors could truly be considered risk factors. Family history of premature coronary heart disease, which was not collected in the PMS, could not be counted as a CV risk factor. As a result, some patients who had coronary heart disease may not have been classified as having CV risk factors. However, it was possible that the impact was not so high, since the proportion with “Yes” was only about 10% in the ORAL Surveillance study. Furthermore, unknown values affected each model selection; however, the results after performing multiple imputation as a sensitivity analysis did not change the current interpretation. Nevertheless, there were few obese Japanese patients with RA, and if the missing HDL data were due to levels not being considered particularly concerning, the distribution of CV risk factors may still be somewhat accurately represented in our findings.

## 5 Conclusions

This post hoc analysis showed that the IRs of AEs (MACE, malignancies, and SI) were higher in older patients with RA treated with tofacitinib. Furthermore, the risks of MACE and SI were higher in patients with CV risk factors than those without. The risk of MACE was notably high in patients with at least 1 CV risk factor. For malignancies and SI, the increased risks might have been influenced by specific CV risk factors, some of which are recognized not only as CV risk factors but also as individual risk factors for these AEs. Most of the risk factors for MACE, malignancies, and SI identified in this study were consistent with previously established risk factors. Some additional statistically significant risk factors were identified but could not be considered new risk factors for AEs due to their established correlation with known risk factors such as older age and high RA disease activity.

Overall, this post hoc analysis of the all‐case PMS study provides valuable insight into the impact of age and various risk factors associated with tofacitinib‐related AEs in patients with RA in real‐world clinical practice settings in Japan.

## Author Contributions

All authors contributed to the conception and design of the study. Y.E., K.Y., T.H., and M.H. were involved in data analysis. All authors were involved in the interpretation of the data, were involved in drafting the article or revising it critically for important intellectual content, and all authors approved the final version to be submitted for publication.

## Funding

This work was supported by Pfizer.

## Conflicts of Interest

K.Y. has received grants and/or research support and speakers' fees and/or honoraria from AbbVie, Actelion Japan, Asahi Kasei, Astellas, Ayumi, Boehringer Ingelheim Japan, Bristol Myers Squibb, Chugai, Daiichi Sankyo, Eisai, Eli Lilly Japan, Gilead Sciences, GSK, Hisamitsu, Janssen, Japan Tobacco Inc., Mitsubishi Tanabe Pharma, MSD, Nippon Kayaku, Nippon Shinyaku, Ono, Otsuka, Pfizer Inc., Sanofi, Takeda, and Teijin. T.H. and M.H. are employees and shareholders of Pfizer Japan Inc. Y.E. is an employee and shareholder of Pfizer R&D Japan.

## Supporting information


**Table S1:** Definitions of corresponding AEs of interest associated with tofacitinib.
**Table S2:** Patients' baseline characteristics and demographic details stratified by each combination of age and the number of CV risk factors.
**Table S3:** Patients' background factors for the incidence of MACE.
**Table S4:** Patients' background factors for the incidence of malignancies.
**Table S5:** Patients' background factors for the incidence of SI.

**TABLE S6:1** Incidence of MACE stratified by each combination of age and the number of CV risk factors after performing multiple imputation.
**Table S6:**2 Incidence of malignancy stratified by each combination of age and the number of CV risk factors after performing multiple imputation.
**Table S6:**3 Incidence of serious infection stratified by each combination of age and the number of CV risk factors after performing multiple imputation.
**Table S7:**1 Incidence of MACE by CV risk factors after performing multiple imputation.
**Table S7:**2 Incidence of malignancy by CV risk factors after performing multiple imputation.
**Table S7:**3 Incidence of serious infection by CV risk factors after performing multiple imputation.
**Table S8:**1 Incidence of MACE by other demographic factors after performing multiple imputation.
**Table S8:**2 Incidence of malignancy by other demographic factors after performing multiple imputation.
**Table S8:**3 Incidence of serious infection by other demographic factors after performing multiple imputation.
**Table S9:**1 Incidence of malignancy stratified by each combination of age and the number of CV risk factors after changing the definition of exposure period as the on‐treatment period plus 90 days.
**Table S9:**2 Incidence of malignancy by CV risk factors after changing the definition of exposure period as the on‐treatment period plus 90 days.
**Table S9:**3 Incidence of malignancy by other demographic factors after changing the definition of exposure period as the on‐treatment period plus 90 days.

## Data Availability

Upon request and subject to review, Pfizer will provide the data that support the findings of this study. Subject to certain criteria, conditions, and exceptions, Pfizer may also provide access to the related individual de‐identified participant data. See https://www.pfizer.com/science/clinical‐trials/trial‐data‐and‐results for more information.

## References

[1] T. A. Simon , A. Thompson , K. K. Gandhi , M. C. Hochberg , and S. Suissa , “Incidence of Malignancy in Adult Patients With Rheumatoid Arthritis: A Meta‐Analysis,” Arthritis Research & Therapy 17 (2015): 212. 26271620 10.1186/s13075-015-0728-9 PMC4536786

[2] J. A. Singh , K. G. Saag , S. L. Bridges, Jr. , et al., “2015 American College of Rheumatology Guideline for the Treatment of Rheumatoid Arthritis,” Arthritis Care & Research (Hoboken) 68 (2016): 1–25. 10.1002/acr.22783 26545825

[3] M. Kojima , T. Nakayama , K. Tsutani , et al., “Epidemiological Characteristics of Rheumatoid Arthritis in Japan: Prevalence Estimates Using a Nationwide Population‐Based Questionnaire Survey,” Modern Rheumatology 30 (2020): 941–947. 31625435 10.1080/14397595.2019.1682776

[4] “XELJANZ (Tofacitinib Citrate),” accessed May 6, 2025, https://www.pfizer.com/news/press‐release/press‐release‐detail/xeljanz_tofacitinib_citrate_approved_in_japan_for_the_treatment_of_adults_with_rheumatoid_arthritis_ra.

[5] R. Fleischmann , M. Cutolo , M. C. Genovese , et al., “Phase IIb Dose‐Ranging Study of the Oral JAK Inhibitor Tofacitinib (CP‐690,550) or Adalimumab Monotherapy Versus Placebo in Patients With Active Rheumatoid Arthritis With an Inadequate Response to Disease‐Modifying Antirheumatic Drugs,” Arthritis and Rheumatism 64 (2012): 617–629. 21952978 10.1002/art.33383

[6] J. M. Kremer , B. J. Bloom , F. C. Breedveld , et al., “The Safety and Efficacy of a JAK Inhibitor in Patients With Active Rheumatoid Arthritis: Results of a Double‐Blind, Placebo‐Controlled Phase IIa Trial of Three Dosage Levels of CP‐690,550 Versus Placebo,” Arthritis and Rheumatism 60 (2009): 1895–1905. 19565475 10.1002/art.24567

[7] G. R. Burmester , R. Blanco , C. Charles‐Schoeman , et al., “Tofacitinib (CP‐690,550) in Combination With Methotrexate in Patients With Active Rheumatoid Arthritis With an Inadequate Response to Tumour Necrosis Factor Inhibitors: A Randomised Phase 3 Trial,” Lancet 381 (2013): 451–460. 23294500 10.1016/S0140-6736(12)61424-X

[8] J. M. Kremer , S. Cohen , B. E. Wilkinson , et al., “A Phase IIb Dose‐Ranging Study of the Oral JAK Inhibitor Tofacitinib (CP‐690,550) Versus Placebo in Combination With Background Methotrexate in Patients With Active Rheumatoid Arthritis and an Inadequate Response to Methotrexate Alone,” Arthritis and Rheumatism 64 (2012): 970–981. 22006202 10.1002/art.33419

[9] R. Fleischmann , J. Kremer , J. Cush , et al., “Placebo‐Controlled Trial of Tofacitinib Monotherapy in Rheumatoid Arthritis,” New England Journal of Medicine 367 (2012): 495–507. 22873530 10.1056/NEJMoa1109071

[10] R. Fleischmann , E. Mysler , S. Hall , et al., “Efficacy and Safety of Tofacitinib Monotherapy, Tofacitinib With Methotrexate, and Adalimumab With Methotrexate in Patients With Rheumatoid Arthritis (ORAL Strategy): A Phase 3b/4, Double‐Blind, Head‐To‐Head, Randomised Controlled Trial,” Lancet 390 (2017): 457–468. 28629665 10.1016/S0140-6736(17)31618-5

[11] Y. Tanaka , T. Takeuchi , H. Yamanaka , H. Nakamura , S. Toyoizumi , and S. Zwillich , “Effi Cacy and Safety of Tofacitinib as Monotherapy in Japanese Patients With Active Rheumatoid Arthritis: A 12‐Week, Randomized, Phase 2 Study,” Modern Rheumatology 25 (2015): 514–521. 25496464 10.3109/14397595.2014.995875 PMC4819568

[12] Y. Tanaka , M. Suzuki , H. Nakamura , S. Toyoizumi , and S. H. Zwillich , “Phase II Study of Tofacitinib (CP‐690,550) Combined With Methotrexate in Patients With Rheumatoid Arthritis and an Inadequate Response to Methotrexate,” Arthritis Care & Research 63 (2011): 1150–1158. 10.1002/acr.20494 21584942

[13] D. Van Der Heijde , Y. Tanaka , R. Fleischmann , et al., “Tofacitinib (CP‐690,550) in Patients With Rheumatoid Arthritis Receiving Methotrexate: Twelve‐Month Data From a Twenty‐Four‐Month Phase III Randomized Radiographic Study,” Arthritis & Rheumatism 65 (2013): 559–570. 23348607 10.1002/art.37816

[14] D. van der Heijde , V. Strand , Y. Tanaka , et al., “Tofacitinib in Combination With Methotrexate in Patients With Rheumatoid Arthritis: Clinical Efficacy, Radiographic, and Safety Outcomes From a Twenty‐Four–Month, Phase III Study,” Arthritis and Rheumatology 71 (2019): 878–891. 30666826 10.1002/art.40803 PMC6593705

[15] J. Kremer , Z. G. Li , S. Hall , et al., “Tofacitinib in Combination With Nonbiologic Disease‐Modifying Antirheumatic Drugs in Patients With Active Rheumatoid Arthritis: A Randomized Trial,” Annals of Internal Medicine 159 (2013): 253–261. 24026258 10.7326/0003-4819-159-4-201308200-00006

[16] R. F. van Vollenhoven , R. Fleischmann , S. Cohen , et al., “Tofacitinib or Adalimumab Versus Placebo in Rheumatoid Arthritis,” New England Journal of Medicine 367 (2012): 508–519. 22873531 10.1056/NEJMoa1112072

[17] E. B. Lee , R. Fleischmann , S. Hall , et al., “Tofacitinib Versus Methotrexate in Rheumatoid Arthritis,” New England Journal of Medicine 370 (2014): 2377–2386. 24941177 10.1056/NEJMoa1310476

[18] H. Yamanaka , Y. Tanaka , T. Takeuchi , et al., “Tofacitinib, an Oral Janus Kinase Inhibitor, as Monotherapy or With Background Methotrexate, in Japanese Patients With Rheumatoid Arthritis: An Open‐Label, Long‐Term Extension Study,” Arthritis Research & Therapy 18 (2016): 34. 26818974 10.1186/s13075-016-0932-2 PMC4730592

[19] J. Wollenhaupt , J. Silverfield , E. B. Lee , et al., “Safety and Efficacy of Tofacitinib, an Oral Janus Kinase Inhibitor, for the Treatment of Rheumatoid Arthritis in Open‐Label, Long Term Extension Studies,” Journal of Rheumatology 41 (2014): 837–852. 24692527 10.3899/jrheum.130683

[20] J. Wollenhaupt , E. B. Lee , J. R. Curtis , et al., “Safety and Efficacy of Tofacitinib for up to 9.5 Years in the Treatment of Rheumatoid Arthritis: Final Results of a Global, Open‐Label, Long‐Term Extension Study,” Arthritis Research & Therapy 21 (2019): 89. 30953540 10.1186/s13075-019-1866-2 PMC6451219

[21] A. R. Balanescu , G. Citera , V. Pascual‐Ramos , et al., “Infections in Patients With Rheumatoid Arthritis Receiving Tofacitinib Versus Tumour Necrosis Factor Inhibitors: Results From the Open‐Label, Randomised Controlled ORAL Surveillance Trial,” Annals of the Rheumatic Diseases 81 (2022): 1491–1503. 35922124 10.1136/ard-2022-222405 PMC9606533

[22] J. R. Curtis , K. Yamaoka , Y. H. Chen , et al., “Malignancy Risk With Tofacitinib Versus TNF Inhibitors in Rheumatoid Arthritis: Results From the Open‐Label, Randomised Controlled ORAL Surveillance Trial,” Annals of the Rheumatic Diseases 82 (2023): 331–343. 36600185 10.1136/ard-2022-222543 PMC9933177

[23] L. E. Kristensen , S. Danese , A. Yndestad , et al., “Identification of Two Tofacitinib Subpopulations With Different Relative Risk Versus TNF Inhibitors: An Analysis of the Open Label, Randomised Controlled Study ORAL Surveillance,” Annals of the Rheumatic Diseases 82 (2023): 901–910. 36931693 10.1136/ard-2022-223715 PMC10314011

[24] M. Kuwana , N. Sugiyama , S. Momohara , et al., “Six‐Month Safety and Effectiveness of Tofacitinib in Patients With Rheumatoid Arthritis in Japan: Interim Analysis of Post‐Marketing Surveillance,” Modern Rheumatology 34 (2024): 272–286. 37405710 10.1093/mr/road063

[25] S. R. Ytterberg , D. L. Bhatt , T. R. Mikuls , et al., “Cardiovascular and Cancer Risk With Tofacitinib in Rheumatoid Arthritis,” New England Journal of Medicine 386 (2022): 316–326. 35081280 10.1056/NEJMoa2109927

[26] C. Charles‐Schoeman , M. H. Buch , M. Dougados , et al., “Risk of Major Adverse Cardiovascular Events With Tofacitinib Versus Tumour Necrosis Factor Inhibitors in Patients With Rheumatoid Arthritis With or Without a History of Atherosclerotic Cardiovascular Disease: A Post Hoc Analysis From ORAL Surveillance,” Annals of the Rheumatic Diseases 82 (2022): 119–129. 36137735 10.1136/ard-2022-222259 PMC9811099

[27] K. Yamaoka , N. Sugiyama , M. Hoshi , J. Y. Jo , K. Shin , and T. Hirano , “Risk Factors Associated With Major Adverse Cardiovascular Events and Malignancies in Patients With Rheumatoid Arthritis in a Real‐World Setting in Japan,” International Journal of Rheumatic Diseases 27 (2024): e1544.8. 10.1111/1756-185X.15448 PMC11626545 39648942

[28] M. Kuwana , N. Sugiyama , S. Momohara , et al., “Three‐Year Safety and Effectiveness of Tofacitinib in Patients With Rheumatoid Arthritis in Japan: Final Analysis of an All‐Case Post‐Marketing Surveillance Study,” Modern Rheumatology 35 (2025): roaf017. 10.1093/mr/roaf017 40053522

[29] D. B. Rubin , Multiple Imputation for Nonresponse in Surveys (John Wiley & Sons, 1987).

[30] G. A. Karpouzas , Z. Szekanecz , E. Baecklund , et al., “Rheumatoid Arthritis Disease Activity and Adverse Events in Patients Receiving Tofacitinib or Tumor Necrosis Factor Inhibitors: A Post Hoc Analysis of ORAL Surveillance,” Therapeutic Advances in Musculoskeletal Disease 15 (2023): 1759720X231201047. 10.1177/1759720X231201047 PMC10629315 37942277

