# Supplementary material for: Risk Factors for Major Adverse Cardiovascular Events, Malignancies, and Serious Infections With Tofacitinib in Rheumatoid Arthritis: Post Hoc Analysis of a 3‐Year J‐Post‐Marketing Surveillance
Source: Int J Rheum Dis. 2026 Feb 9;29(2):e70572. doi: 10.1111/1756-185x.70572 (PMC12886613; doi:10.1111/1756-185x.70572)
Supplement: Supplementary file 1 — Table S1: Definitions of corresponding AEs of interest associated with tofacitinib. Table S2: Patients' baseline characteristics and demographic details stratified by each combination of age and the number of CV risk factors. Table S3: Patients' background factors for the incidence of MACE. Table S4: Patients' background factors for the incidence of malignancies. Table S5: Patients' background factors for the incidence of SI. [file APL-29-e70572-s002.docx]

**SUPPLEMENTARY FILE**

**TABLE S1** Definitions of corresponding AEs of interest associated with tofacitinib

| **AEs^a^** | **Definitions of AEs** |
| --- | --- |
| MACE | MACE corresponds to the following:   1. CV death    - Death due to acute myocardial infarction    - Sudden cardiac death    - Death due to heart failure    - Death due to stroke    - Death due to CV procedures    - Death due to CV haemorrhage    - Death due to other CV causes, such as peripheral artery disease 2. Non-fatal myocardial infarction 3. Non-fatal stroke of any classification, including reversible focal neurological defects with imaging evidence of a new cerebral lesion consistent with ischaemia or haemorrhage |
| Malignancies | Tumours with a definitive diagnosis among malignant tumours, including lymphoma |
| SI | SI included infections corresponding to the following:  1. Resulting in death or life-threatening  2. Resulting in disability or may lead to disability  3. Requiring inpatient hospitalisation or prolongation of hospitalisation  4. Serious in accordance with the above |

^a^Coded using MedDRA/J, version 23.1.

AE, adverse event; CV, cardiovascular; MACE, major adverse cardiovascular events; MedDRA/J, English–Japanese Medical Dictionary for Regulatory Activities; SI, serious infection.

**TABLE S2** Patients’ baseline characteristics and demographic details stratified by each combination of age and the number of CV risk factors

| **Characteristics** | | **Safety analysis set (N=7,021)** | **<50 years** | | **≥50 to <65 years** | | **≥65 years** | |
| --- | --- | --- | --- | --- | --- | --- | --- | --- |
|  |  |  | **0 CV risk factor (n=844)** | **≥1 CV risk factors**  **(n=268)** | **0 CV risk factor (n=1,441)** | **≥1 CV risk factors (n=843)** | **0 CV risk factor (n=1,901)** | **≥1 CV risk factors (n=1,724)** |
| Age (years) | <50 | 1,112 (15.8) | 844 (100.0) | 268 (100.0) | 0 (0.0) | 0 (0.0) | 0 (0.0) | 0 (0.0) |
|  | ≥50 to <65 | 2,284 (32.5) | 0 (0.0) | 0 (0.0) | 1,441 (100.0) | 843 (100.0) | 0 (0.0) | 0 (0.0) |
|  | ≥65 | 3,625 (51.6) | 0 (0.0) | 0 (0.0) | 0 (0.0) | 0 (0.0) | 1,901 (100.0) | 1,724 (100.0) |
| Sex^a^ | Male | 1,423 (20.3) | 91 (10.8) | 97 (36.2) | 203 (14.1) | 271 (32.1) | 300 (15.8) | 461 (26.7) |
|  | Female | 5,598 (79.7) | 753 (89.2) | 171 (63.8) | 1,238 (85.9) | 572 (67.9) | 1,601 (84.2) | 1,263 (73.3) |
| Weight (kg) | <50 | 1,955 (27.8) | 220 (26.1) | 36 (13.4) | 355 (24.6) | 144 (17.1) | 697 (36.7) | 503 (29.2) |
|  | ≥50 to <60 | 1,979 (28.2) | 248 (29.4) | 66 (24.6) | 433 (30.0) | 212 (25.1) | 501 (26.4) | 519 (30.1) |
|  | ≥60 to <70 | 1,084 (15.4) | 101 (12.0) | 51 (19.0) | 215 (14.9) | 203 (24.1) | 198 (10.4) | 316 (18.3) |
|  | ≥70 | 594 (8.5) | 75 (8.9) | 78 (29.1) | 107 (7.4) | 151 (17.9) | 63 (3.3) | 120 (7.0) |
|  | Unknown | 1,409 (20.1) | 200 (23.7) | 37 (13.8) | 331 (23.0) | 133 (15.8) | 442 (23.3) | 266 (15.4) |
| Duration of disease (years) | <2 | 787 (11.2) | 105 (12.4) | 46 (17.2) | 158 (11.0) | 112 (13.3) | 205 (10.8) | 161 (9.3) |
|  | ≥2 to <5 | 1,052 (15.0) | 167 (19.8) | 48 (17.9) | 203 (14.1) | 130 (15.4) | 252 (13.3) | 252 (14.6) |
|  | ≥5 to <10 | 1,394 (19.9) | 196 (23.2) | 64 (23.9) | 294 (20.4) | 171 (20.3) | 355 (18.7) | 314 (18.2) |
|  | ≥10 to <20 | 1,841 (26.2) | 194 (23.0) | 65 (24.3) | 420 (29.1) | 241 (28.6) | 487 (25.6) | 434 (25.2) |
|  | ≥20 | 1,133 (16.1) | 72 (8.5) | 20 (7.5) | 204 (14.2) | 124 (14.7) | 352 (18.5) | 361 (20.9) |
|  | Unknown | 814 (11.6) | 110 (13.0) | 25 (9.3) | 162 (11.2) | 65 (7.7) | 250 (13.2) | 202 (11.7) |
| Stage of RA | I-II | 3,109 (44.3) | 465 (55.1) | 171 (63.8) | 646 (44.8) | 396 (47.0) | 773 (40.7) | 658 (38.2) |
|  | III | 1,767 (25.2) | 198 (23.5) | 48 (17.9) | 369 (25.6) | 205 (24.3) | 498 (26.2) | 449 (26.0) |
|  | IV | 2,106 (30.0) | 174 (20.6) | 47 (17.5) | 422 (29.3) | 237 (28.1) | 619 (32.6) | 607 (35.2) |
|  | Unknown | 39 (0.6) | 7 (0.8) | 2 (0.7) | 4 (0.3) | 5 (0.6) | 11 (0.6) | 10 (0.6) |
| Class of RA | 1-2 | 5,276 (75.1) | 714 (84.6) | 226 (84.3) | 1,152 (79.9) | 648 (76.9) | 1,350 (71.0) | 1,186 (68.8) |
|  | 3 | 1,544 (22.0) | 116 (13.7) | 34 (12.7) | 265 (18.4) | 177 (21.0) | 485 (25.5) | 467 (27.1) |
|  | 4 | 166 (2.4) | 7 (0.8) | 6 (2.2) | 20 (1.4) | 14 (1.7) | 56 (2.9) | 63 (3.7) |
|  | Unknown | 35 (0.5) | 7 (0.8) | 2 (0.7) | 4 (0.3) | 4 (0.5) | 10 (0.5) | 8 (0.5) |
| Presence of extra-articular disease associated with RA | No | 6,748 (96.1) | 844 (100.0) | 250 (93.3) | 1,441 (100.0) | 770 (91.3) | 1,900 (99.9) | 1,543 (89.5) |
|  | Yes | 272 (3.9) | 0 (0.0) | 18 (6.7) | 0 (0.0) | 73 (8.7) | 0 (0.0) | 181 (10.5) |
|  | Unknown | 1 (0.0) | 0 (0.0) | 0 (0.0) | 0 (0.0) | 0 (0.0) | 1 (0.1) | 0 (0.0) |
| Smoking history | No | 4,833 (68.8) | 657 (77.8) | 95 (35.4) | 1,086 (75.4) | 437 (51.8) | 1,403 (73.8) | 1,155 (67.0) |
|  | Yes | 662 (9.4) | 0 (0.0) | 149 (55.6) | 0 (0.0) | 284 (33.7) | 0 (0.0) | 229 (13.3) |
|  | Unknown | 1,526 (21.7) | 187 (22.2) | 24 (9.0) | 355 (24.6) | 122 (14.5) | 498 (26.2) | 340 (19.7) |
| HDL <40 mg/dL during treatment | No | 1,274 (18.1) | 133 (15.8) | 56 (20.9) | 252 (17.5) | 174 (20.6) | 347 (18.3) | 312 (18.1) |
|  | Yes | 130 (1.9) | 0 (0.0) | 21 (7.8) | 0 (0.0) | 36 (4.3) | 0 (0.0) | 73 (4.2) |
|  | Unknown | 5,617 (80.0) | 711 (84.2) | 191 (71.3) | 1,189 (82.5) | 633 (75.1) | 1,554 (81.7) | 1,339 (77.7) |
| Concomitant hypertension | No | 5,325 (75.8) | 844 (100.0) | 191 (71.3) | 1,441 (100.0) | 385 (45.7) | 1,900 (99.9) | 564 (32.7) |
|  | Yes | 1,695 (24.1) | 0 (0.0) | 77 (28.7) | 0 (0.0) | 458 (54.3) | 0 (0.0) | 1,160 (67.3) |
|  | Unknown | 1 (0.0) | 0 (0.0) | 0 (0.0) | 0 (0.0) | 0 (0.0) | 1 (0.1) | 0 (0.0) |
| Concomitant diabetes mellitus | No | 6,227 (88.7) | 844 (100.0) | 235 (87.7) | 1,441 (100.0) | 647 (76.7) | 1,900 (99.9) | 1,160 (67.3) |
|  | Yes | 793 (11.3) | 0 (0.0) | 33 (12.3) | 0 (0.0) | 196 (23.3) | 0 (0.0) | 564 (32.7) |
|  | Unknown | 1 (0.0) | 0 (0.0) | 0 (0.0) | 0 (0.0) | 0 (0.0) | 1 (0.1) | 0 (0.0) |
| Status of methotrexate use (dose at baseline; mg/week)^b^ | No | 2,846 (40.5) | 232 (27.5) | 81 (30.2) | 444 (30.8) | 305 (36.2) | 838 (44.1) | 946 (54.9) |
|  | ≤8 | 2,116 (30.1) | 244 (28.9) | 66 (24.6) | 481 (33.4) | 228 (27.0) | 637 (33.5) | 460 (26.7) |
|  | >8 | 2,058 (29.3) | 368 (43.6) | 121 (45.1) | 516 (35.8) | 310 (36.8) | 426 (22.4) | 317 (18.4) |
|  | Unknown | 1 (0.0) | 0 (0.0) | 0 (0.0) | 0 (0.0) | 0 (0.0) | 0 (0.0) | 1 (0.1) |
| Status of oral steroid use (dose at baseline; mg/day) | No | 3,159 (45.0) | 371 (44.0) | 97 (36.2) | 727 (50.5) | 351 (41.6) | 884 (46.5) | 729 (42.3) |
|  | <2.5 | 991 (14.1) | 96 (11.4) | 27 (10.1) | 197 (13.7) | 108 (12.8) | 297 (15.6) | 266 (15.4) |
|  | ≥2.5 to <5 | 1,815 (25.9) | 209 (24.8) | 78 (29.1) | 340 (23.6) | 222 (26.3) | 493 (25.9) | 473 (27.4) |
|  | ≥5 | 957 (13.6) | 158 (18.7) | 65 (24.3) | 156 (10.8) | 152 (18.0) | 191 (10.0) | 235 (13.6) |
|  | Unknown | 99 (1.4) | 10 (1.2) | 1 (0.4) | 21 (1.5) | 10 (1.2) | 36 (1.9) | 21 (1.2) |
| Initial tofacitinib dose (mg/day) | 5 | 2,343 (33.4) | 197 (23.3) | 72 (26.9) | 390 (27.1) | 222 (26.3) | 747 (39.3) | 715 (41.5) |
|  | 10 | 4,640 (66.1) | 644 (76.3) | 196 (73.1) | 1,040 (72.2) | 618 (73.3) | 1,143 (60.1) | 999 (57.9) |
|  | Unknown | 38 (0.5) | 3 (0.4) | 0 (0.0) | 11 (0.8) | 3 (0.4) | 11 (0.6) | 10 (0.6) |
| History of coronary artery disease | No | 6,922 (98.6) | 844 (100.0) | 263 (98.1) | 1,441 (100.0) | 828 (98.2) | 1,900 (99.9) | 1,646 (95.5) |
|  | Yes | 98 (1.4) | 0 (0.0) | 5 (1.9) | 0 (0.0) | 15 (1.8) | 0 (0.0) | 78 (4.5) |
|  | Unknown | 1 (0.0) | 0 (0.0) | 0 (0.0) | 0 (0.0) | 0 (0.0) | 1 (0.1) | 0 (0.0) |
| History of infection | No | 6,061 (86.3) | 771 (91.4) | 235 (87.7) | 1,270 (88.1) | 706 (83.7) | 1,652 (86.9) | 1,427 (82.8) |
|  | Yes | 959 (13.7) | 73 (8.6) | 33 (12.3) | 171 (11.9) | 137 (16.3) | 248 (13.0) | 297 (17.2) |
|  | Unknown | 1 (0.0) | 0 (0.0) | 0 (0.0) | 0 (0.0) | 0 (0.0) | 1 (0.1) | 0 (0.0) |
| History of HZ | No | 6,659 (94.8) | 816 (96.7) | 255 (95.1) | 1,367 (94.9) | 805 (95.5) | 1,813 (95.4) | 1,603 (93.0) |
|  | Yes | 361 (5.1) | 28 (3.3) | 13 (4.9) | 74 (5.1) | 38 (4.5) | 87 (4.6) | 121 (7.0) |
|  | Unknown | 1 (0.0) | 0 (0.0) | 0 (0.0) | 0 (0.0) | 0 (0.0) | 1 (0.1) | 0 (0.0) |
| History of malignancies | No | 6,737 (96.0) | 831 (98.5) | 263 (98.1) | 1,397 (96.9) | 820 (97.3) | 1,810 (95.2) | 1,616 (93.7) |
|  | Yes | 283 (4.0) | 13 (1.5) | 5 (1.9) | 44 (3.1) | 23 (2.7) | 90 (4.7) | 108 (6.3) |
|  | Unknown | 1 (0.0) | 0 (0.0) | 0 (0.0) | 0 (0.0) | 0 (0.0) | 1 (0.1) | 0 (0.0) |
| History of CV disease | No | 6,628 (94.4) | 832 (98.6) | 255 (95.1) | 1,409 (97.8) | 792 (94.0) | 1,802 (94.8) | 1,538 (89.2) |
|  | Yes | 392 (5.6) | 12 (1.4) | 13 (4.9) | 32 (2.2) | 51 (6.0) | 98 (5.2) | 186 (10.8) |
|  | Unknown | 1 (0.0) | 0 (0.0) | 0 (0.0) | 0 (0.0) | 0 (0.0) | 1 (0.1) | 0 (0.0) |
| History of lung disorder | No | 6,694 (95.3) | 837 (99.2) | 255 (95.1) | 1,427 (99.0) | 764 (90.6) | 1,870 (98.4) | 1,541 (89.4) |
|  | Yes | 326 (4.6) | 7 (0.8) | 13 (4.9) | 14 (1.0) | 79 (9.4) | 30 (1.6) | 183 (10.6) |
|  | Unknown | 1 (0.0) | 0 (0.0) | 0 (0.0) | 0 (0.0) | 0 (0.0) | 1 (0.1) | 0 (0.0) |
| History of interstitial pneumonia | No | 6,785 (96.6) | 844 (100.0) | 256 (95.5) | 1,441 (100.0) | 780 (92.5) | 1,899 (99.9) | 1,565 (90.8) |
|  | Yes | 235 (3.3) | 0 (0.0) | 12 (4.5) | 0 (0.0) | 63 (7.5) | 1 (0.1) | 159 (9.2) |
|  | Unknown | 1 (0.0) | 0 (0.0) | 0 (0.0) | 0 (0.0) | 0 (0.0) | 1 (0.1) | 0 (0.0) |
| History of metabolic abnormality | No | 6,923 (98.6) | 839 (99.4) | 265 (98.9) | 1,427 (99.0) | 830 (98.5) | 1,868 (98.3) | 1,694 (98.3) |
|  | Yes | 96 (1.4) | 5 (0.6) | 2 (0.7) | 14 (1.0) | 13 (1.5) | 32 (1.7) | 30 (1.7) |
|  | Unknown | 2 (0.0) | 0 (0.0) | 1 (0.4) | 0 (0.0) | 0 (0.0) | 1 (0.1) | 0 (0.0) |
| History of tacrolimus use^c^ | No | 5,957 (84.8) | 716 (84.8) | 219 (81.7) | 1,225 (85.0) | 709 (84.1) | 1,649 (86.7) | 1,439 (83.5) |
|  | Yes | 967 (13.8) | 115 (13.6) | 48 (17.9) | 199 (13.8) | 122 (14.5) | 225 (11.8) | 258 (15.0) |
|  | Unknown | 97 (1.4) | 13 (1.5) | 1 (0.4) | 17 (1.2) | 12 (1.4) | 27 (1.4) | 27 (1.6) |
| History of biological agent use | No | 3,329 (47.4) | 399 (47.3) | 123 (45.9) | 695 (48.2) | 372 (44.1) | 957 (50.3) | 783 (45.4) |
|  | Yes | 3,692 (52.6) | 445 (52.7) | 145 (54.1) | 746 (51.8) | 471 (55.9) | 944 (49.7) | 941 (54.6) |
|  | Unknown | 0 (0.0) | 0 (0.0) | 0 (0.0) | 0 (0.0) | 0 (0.0) | 0 (0.0) | 0 (0.0) |
| Family history of malignancies (including lymphoma) | No | 4,003 (57.0) | 524 (62.1) | 156 (58.2) | 827 (57.4) | 470 (55.8) | 1,080 (56.8) | 946 (54.9) |
|  | Yes | 533 (7.6) | 54 (6.4) | 32 (11.9) | 117 (8.1) | 78 (9.3) | 109 (5.7) | 143 (8.3) |
|  | Unknown | 2,485 (35.4) | 266 (31.5) | 80 (29.9) | 497 (34.5) | 295 (35.0) | 712 (37.5) | 635 (36.8) |
| Complication (infection) | No | 6,726 (95.8) | 821 (97.3) | 257 (95.9) | 1,388 (96.3) | 802 (95.1) | 1,829 (96.2) | 1,629 (94.5) |
|  | Yes | 294 (4.2) | 23 (2.7) | 11 (4.1) | 53 (3.7) | 41 (4.9) | 71 (3.7) | 95 (5.5) |
|  | Unknown | 1 (0.0) | 0 (0.0) | 0 (0.0) | 0 (0.0) | 0 (0.0) | 1 (0.1) | 0 (0.0) |
| Complication (HZ) | No | 7,015 (99.9) | 844 (100.0) | 267 (99.6) | 1,441 (100.0) | 843 (100.0) | 1,898 (99.8) | 1,722 (99.9) |
|  | Yes | 5 (0.1) | 0 (0.0) | 1 (0.4) | 0 (0.0) | 0 (0.0) | 2 (0.1) | 2 (0.1) |
|  | Unknown | 1 (0.0) | 0 (0.0) | 0 (0.0) | 0 (0.0) | 0 (0.0) | 1 (0.1) | 0 (0.0) |
| Complication (malignancies) | No | 6,980 (99.4) | 841 (99.6) | 266 (99.3) | 1,437 (99.7) | 840 (99.6) | 1,887 (99.3) | 1,709 (99.1) |
|  | Yes | 40 (0.6) | 3 (0.4) | 2 (0.7) | 4 (0.3) | 3 (0.4) | 13 (0.7) | 15 (0.9) |
|  | Unknown | 1 (0.0) | 0 (0.0) | 0 (0.0) | 0 (0.0) | 0 (0.0) | 1 (0.1) | 0 (0.0) |
| Complication (CV disease) | No | 5,103 (72.7) | 832 (98.6) | 187 (69.8) | 1,406 (97.6) | 366 (43.4) | 1,802 (94.8) | 510 (29.6) |
|  | Yes | 1,917 (27.3) | 12 (1.4) | 81 (30.2) | 35 (2.4) | 477 (56.6) | 98 (5.2) | 1,214 (70.4) |
|  | Unknown | 1 (0.0) | 0 (0.0) | 0 (0.0) | 0 (0.0) | 0 (0.0) | 1 (0.1) | 0 (0.0) |
| Complication (lung disorder) | No | 6,288 (89.6) | 816 (96.7) | 254 (94.8) | 1,344 (93.3) | 759 (90.0) | 1,712 (90.1) | 1,403 (81.4) |
|  | Yes | 732 (10.4) | 28 (3.3) | 14 (5.2) | 97 (6.7) | 84 (10.0) | 188 (9.9) | 321 (18.6) |
|  | Unknown | 1 (0.0) | 0 (0.0) | 0 (0.0) | 0 (0.0) | 0 (0.0) | 1 (0.1) | 0 (0.0) |
| Complication (interstitial pneumonia) | No | 6,563 (93.5) | 826 (97.9) | 260 (97.0) | 1,386 (96.2) | 799 (94.8) | 1,779 (93.6) | 1,513 (87.8) |
|  | Yes | 457 (6.5) | 18 (2.1) | 8 (3.0) | 55 (3.8) | 44 (5.2) | 121 (6.4) | 211 (12.2) |
|  | Unknown | 1 (0.0) | 0 (0.0) | 0 (0.0) | 0 (0.0) | 0 (0.0) | 1 (0.1) | 0 (0.0) |
| Complication (metabolic abnormality) | No | 5,097 (72.6) | 785 (93.0) | 186 (69.4) | 1,225 (85.0) | 450 (53.4) | 1,627 (85.6) | 824 (47.8) |
|  | Yes | 1,922 (27.4) | 59 (7.0) | 81 (30.2) | 216 (15.0) | 393 (46.6) | 273 (14.4) | 900 (52.2) |
|  | Unknown | 2 (0.0) | 0 (0.0) | 1 (0.4) | 0 (0.0) | 0 (0.0) | 1 (0.1) | 0 (0.0) |

Data are represented as n (%).

Missing data of covariates were categorised as ‘unknown’.

^a^Excluding unknown cases.

^b^Received at the start of tofacitinib administration.

^c^Within 3 months before the start of the study.

CV, cardiovascular; HDL, high-density lipoprotein; HZ, herpes zoster; RA, rheumatoid arthritis.

**TABLE S3** Patients’ background factors for the incidence of MACE

| **Background factors** | | **Number of patients in the safety analysis set (N)** | **PY** | **Number of patients with AEs**  **n (%)** | **IR/100 PY** | **Univariate analysis** | | | **Multivariate analysis** | |
| --- | --- | --- | --- | --- | --- | --- | --- | --- | --- | --- |
|  |  |  |  |  |  | **HR (95% CI)** | ***p*-value** | **HR (95% CI)** | | ***p*-value** |
| Smoking history | No | 4,833 | 10,750.5 | 50 (1.03) | 0.47 | - | 0.036 | - | | 0.021 |
|  | Yes | 662 | 1,461.3 | 14 (2.11) | 0.96 | 2.03 (1.12-3.68) |  | 2.31 (1.25-4.29) | |  |
|  | Unknown | 1,526 | 3,240.9 | 13 (0.85) | 0.40 | 0.85 (0.46-1.56) |  | 0.94 (0.51-1.74) | |  |
| Concomitant hypertension | No | 5,325 | 11,656.5 | 45 (0.85) | 0.39 | - | 0.003 | - | |  |
|  | Yes | 1,695 | 3,793.1 | 32 (1.89) | 0.84 | 2.20 (1.40-3.47) |  | - | |  |
|  | Unknown | 1 | 3.0 | 0 (0.00) | 0.00 | 0.00 (ND) |  | - | |  |
| HDL <40 mg/dL during treatment | No | 1,274 | 2,862.6 | 14 (1.10) | 0.49 | - | 0.953 | - | |  |
|  | Yes | 130 | 260.8 | 1 (0.77) | 0.38 | 0.77 (0.10-5.86) |  | - | |  |
|  | Unknown | 5,617 | 12,329.2 | 62 (1.10) | 0.50 | 1.04 (0.58-1.85) |  | - | |  |
| Concomitant diabetes mellitus | No | 6,227 | 13,721.3 | 58 (0.93) | 0.42 | - | 0.002 | - | |  |
|  | Yes | 793 | 1,728.3 | 19 (2.40) | 1.10 | 2.58 (1.53-4.33) |  | - | |  |
|  | Unknown | 1 | 3.0 | 0 (0.00) | 0.00 | 0.00 (ND) |  | - | |  |
| History of coronary artery disease | No | 6,922 | 15,246.1 | 73 (1.05) | 0.48 | - | 0.020 | - | |  |
|  | Yes | 98 | 203.6 | 4 (4.08) | 1.96 | 4.23 (1.54-11.57) |  | - | |  |
|  | Unknown | 1 | 3.0 | 0 (0.00) | 0.00 | 0.00 (ND) |  | - | |  |
| Presence of extra-articular disease associated with RA | No | 6,748 | 14,854.9 | 70 (1.04) | 0.47 | - | 0.088 | - | |  |
|  | Yes | 272 | 594.7 | 7 (2.57) | 1.18 | 2.40 (1.10-5.23) |  | - | |  |
|  | Unknown | 1 | 3.0 | 0 (0.00) | 0.00 | 0.00 (ND) |  | - | |  |
| Sex | Male | 1,423 | 3,050.2 | 23 (1.62) | 0.75 | - | 0.026 | - | |  |
|  | Female | 5,598 | 12,402.5 | 54 (0.96) | 0.44 | 0.57 (0.35-0.93) |  | - | |  |
| Age (years) | <50 | 1,112 | 2,512.2 | 6 (0.54) | 0.24 | - | 0.004 | - | |  |
|  | ≥50 to <65 | 2,284 | 5,305.0 | 18 (0.79) | 0.34 | 1.42 (0.56-3.58) |  | - | |  |
|  | ≥65 | 3,625 | 7,635.4 | 53 (1.46) | 0.69 | 2.89 (1.24-6.72) |  | - | |  |
| Weight (kg) | <50 | 1,955 | 4,283.1 | 30 (1.53) | 0.70 | - | 0.076 | - | |  |
|  | ≥50 to <60 | 1,979 | 4,514.0 | 14 (0.71) | 0.31 | 0.45 (0.24-0.85) |  | - | |  |
|  | ≥60 to <70 | 1,084 | 2,386.3 | 16 (1.48) | 0.67 | 0.96 (0.53-1.77) |  | - | |  |
|  | ≥70 | 594 | 1,334.3 | 5 (0.84) | 0.37 | 0.53 (0.21-1.37) |  | - | |  |
|  | Unknown | 1,409 | 2,935.0 | 12 (0.85) | 0.41 | 0.58 (0.30-1.13) |  | - | |  |
| Stage of RA | I-II | 3,109 | 6,682.3 | 26 (0.84) | 0.39 | - | <0.001 | - | | <0.001 |
|  | III | 1,767 | 3,944.6 | 15 (0.85) | 0.38 | 1.00 (0.53-1.88) |  | 0.88 (0.46-1.69) | |  |
|  | IV | 2,106 | 4,771.0 | 33 (1.57) | 0.69 | 1.82 (1.09-3.04) |  | 1.39 (0.80-2.43) | |  |
|  | Unknown | 39 | 54.8 | 3 (7.69) | 5.48 | 12.88  (3.88-42.77) |  | 44.68  (10.42-191.61) | |  |
| Class of RA | 1-2 | 5,276 | 11,655.7 | 44 (0.83) | 0.38 | - | 0.003 | - | | 0.036 |
|  | 3 | 1,544 | 3,412.9 | 28 (1.81) | 0.82 | 2.13 (1.33-3.43) |  | 1.71 (1.03-2.83) | |  |
|  | 4 | 166 | 330.8 | 4 (2.41) | 1.21 | 3.25 (1.17-9.05) |  | 2.32 (0.81-6.63) | |  |
|  | Unknown | 35 | 53.2 | 1 (2.86) | 1.88 | 3.90 (0.53-28.76) |  | 0.13 (0.01-1.47) | |  |
| Duration of disease (years) | <2 | 787 | 1,573.5 | 5 (0.64) | 0.32 | - | 0.113 | - | |  |
|  | ≥2 to <5 | 1,052 | 2,312.5 | 6 (0.57) | 0.26 | 0.78 (0.24-2.56) |  | - | |  |
|  | ≥5 to <10 | 1,394 | 3,090.7 | 18 (1.29) | 0.58 | 1.79 (0.66-4.82) |  | - | |  |
|  | ≥10 to <20 | 1,841 | 4,259.6 | 17 (0.92) | 0.40 | 1.23 (0.45-3.33) |  | - | |  |
|  | ≥20 | 1,133 | 2,556.8 | 18 (1.59) | 0.70 | 2.15 (0.80-5.79) |  | - | |  |
|  | Unknown | 814 | 1,659.5 | 13 (1.60) | 0.78 | 2.32 (0.82-6.51) |  | - | |  |
| History of infection | No | 6,061 | 13,260.2 | 65 (1.07) | 0.49 | - | 0.920 | - | |  |
|  | Yes | 959 | 2,189.5 | 12 (1.25) | 0.55 | 1.14 (0.61-2.11) |  | - | |  |
|  | Unknown | 1 | 3.0 | 0 (0.00) | 0.00 | 0.00 (ND) |  | - | |  |
| History of HZ | No | 6,659 | 14,586.0 | 70 (1.05) | 0.48 | - | 0.394 | - | |  |
|  | Yes | 361 | 863.7 | 7 (1.94) | 0.81 | 1.72 (0.79-3.74) |  | - | |  |
|  | Unknown | 1 | 3.0 | 0 (0.00) | 0.00 | 0.00 (ND) |  | - | |  |
| History of malignancies | No | 6,737 | 14,852.5 | 70 (1.04) | 0.47 | - | 0.058 | - | |  |
|  | Yes | 283 | 597.2 | 7 (2.47) | 1.17 | 2.58 (1.18-5.61) |  | - | |  |
|  | Unknown | 1 | 3.0 | 0 (0.00) | 0.00 | 0.00 (ND) |  | - | |  |
| History of CV disease | No | 6,628 | 14,621.2 | 63 (0.95) | 0.43 | - | <0.001 | - | | <0.001 |
|  | Yes | 392 | 828.4 | 14 (3.57) | 1.69 | 3.92 (2.20-7.00) |  | 3.24 (1.80-5.83) | |  |
|  | Unknown | 1 | 3.0 | 0 (0.00) | 0.00 | 0.00 (ND) |  | 0.00 (ND) | |  |
| History of lung disorder | No | 6,694 | 14,724.9 | 69 (1.03) | 0.47 | - | 0.088 | - | |  |
|  | Yes | 326 | 724.7 | 8 (2.45) | 1.10 | 2.28 (1.10-4.75) |  | - | |  |
|  | Unknown | 1 | 3.0 | 0 (0.00) | 0.00 | 0.00 (ND) |  | - | |  |
| History of interstitial pneumonia | No | 6,785 | 14,929.7 | 70 (1.03) | 0.47 | - | 0.039 | - | |  |
|  | Yes | 235 | 519.9 | 7 (2.98) | 1.35 | 2.75 (1.26-5.98) |  | - | |  |
|  | Unknown | 1 | 3.0 | 0 (0.00) | 0.00 | 0.00 (ND) |  | - | |  |
| History of metabolic abnormality | No | 6,923 | 15,208.0 | 74 (1.07) | 0.49 | - | 0.311 | - | |  |
|  | Yes | 96 | 238.7 | 3 (3.13) | 1.26 | 2.46 (0.78-7.83) |  | - | |  |
|  | Unknown | 2 | 6.0 | 0 (0.00) | 0.00 | 0.00 (ND) |  | - | |  |
| Complication (infection) | No | 6,726 | 14,776.7 | 75 (1.12) | 0.51 | - | 0.748 | - | |  |
|  | Yes | 294 | 673.0 | 2 (0.68) | 0.30 | 0.58 (0.14-2.36) |  | - | |  |
|  | Unknown | 1 | 3.0 | 0 (0.00) | 0.00 | 0.00 (ND) |  | - | |  |
| Complication (HZ) | No | 7,015 | 15,437.3 | 77 (1.10) | 0.50 | - | 1.000 | - | |  |
|  | Yes | 5 | 12.3 | 0 (0.00) | 0.00 | 0.00 (ND) |  | - | |  |
|  | Unknown | 1 | 3.0 | 0 (0.00) | 0.00 | 0.00 (ND) |  | - | |  |
| Complication (malignancies) | No | 6,980 | 15,385.7 | 77 (1.10) | 0.50 | - | 1.000 | - | |  |
|  | Yes | 40 | 63.9 | 0 (0.00) | 0.00 | 0.00 (ND) |  | - | |  |
|  | Unknown | 1 | 3.0 | 0 (0.00) | 0.00 | 0.00 (ND) |  | - | |  |
| Complication (CV disease) | No | 5,103 | 11,190.8 | 37 (0.73) | 0.33 | - | <0.001 | - | | <0.001 |
|  | Yes | 1,917 | 4,258.9 | 40 (2.09) | 0.94 | 2.84 (1.82-4.44) |  | 2.26 (1.43-3.58) | |  |
|  | Unknown | 1 | 3.0 | 0 (0.00) | 0.00 | 0.00 (ND) |  | 0.00 (ND) | |  |
| Complication (lung disorder) | No | 6,288 | 13,882.4 | 65 (1.03) | 0.47 | - | 0.296 | - | |  |
|  | Yes | 732 | 1,567.3 | 12 (1.64) | 0.77 | 1.63 (0.88-3.02) |  | - | |  |
|  | Unknown | 1 | 3.0 | 0 (0.00) | 0.00 | 0.00 (ND) |  | - | |  |
| Complication (interstitial pneumonia) | No | 6,563 | 14,503.7 | 70 (1.07) | 0.48 | - | 0.556 | - | |  |
|  | Yes | 457 | 945.9 | 7 (1.53) | 0.74 | 1.54 (0.71-3.34) |  | - | |  |
|  | Unknown | 1 | 3.0 | 0 (0.00) | 0.00 | 0.00 (ND) |  | - | |  |
| Complication (metabolic abnormality) | No | 5,097 | 11,161.1 | 44 (0.86) | 0.39 | - | 0.016 | - | |  |
|  | Yes | 1,922 | 4,285.6 | 33 (1.72) | 0.77 | 1.94 (1.24-3.05) |  | - | |  |
|  | Unknown | 2 | 6.0 | 0 (0.00) | 0.00 | 0.00 (ND) |  | - | |  |
| Status of methotrexate use (dose at baseline; mg/week) | No | 2,846 | 6,010.6 | 46 (1.62) | 0.77 | - | 0.002 | - | | 0.033 |
|  | ≤8 | 2,116 | 4,682.1 | 20 (0.95) | 0.43 | 0.56 (0.33-0.94) |  | 0.66 (0.39-1.11) | |  |
|  | >8 | 2,058 | 4,756.9 | 11 (0.53) | 0.23 | 0.30 (0.15-0.57) |  | 0.38 (0.19-0.74) | |  |
|  | Unknown | 1 | 3.0 | 0 (0.00) | 0.00 | 0.00 (ND) |  | 0.00 (ND) | |  |
| Status of oral steroid use (dose at baseline; mg/day) | No | 3,159 | 6,984.4 | 28 (0.89) | 0.40 | - | 0.022 | - | |  |
|  | <2.5 | 991 | 2,172.5 | 13 (1.31) | 0.60 | 1.56 (0.81-3.02) |  | - | |  |
|  | ≥2.5 to <5 | 1,815 | 3,993.0 | 15 (0.83) | 0.38 | 0.97 (0.52-1.81) |  | - | |  |
|  | ≥5 | 957 | 2,082.9 | 20 (2.09) | 0.96 | 2.44 (1.37-4.33) |  | - | |  |
|  | Unknown | 99 | 219.9 | 1 (1.01) | 0.45 | 1.07 (0.15-7.89) |  | - | |  |
| History of tacrolimus use | No | 5,957 | 13,045.1 | 57 (0.96) | 0.44 | - | 0.041 | - | |  |
|  | Yes | 967 | 2,219.1 | 19 (1.96) | 0.86 | 1.95 (1.16-3.28) |  | - | |  |
|  | Unknown | 97 | 188.5 | 1 (1.03) | 0.53 | 1.26 (0.17-9.13) |  | - | |  |
| History of biological agent use | No | 3,329 | 7,307.2 | 38 (1.14) | 0.52 | - | 0.746 | - | |  |
|  | Yes | 3,692 | 8,145.4 | 39 (1.06) | 0.48 | 0.93 (0.59-1.45) |  | - | |  |
|  | Unknown | 0 | - | - | - | 0.00 (ND) |  | - | |  |
| Initial tofacitinib dose (mg/day) | 5 | 2,343 | 5,013.2 | 30 (1.28) | 0.60 | - | 0.448 | - | |  |
|  | 10 | 4,640 | 10,353.8 | 47 (1.01) | 0.45 | 0.74 (0.47-1.18) |  | - | |  |
|  | Unknown | 38 | 85.7 | 0 (0.00) | 0.00 | 0.00 (ND) |  | - | |  |
| Family history of malignancies (including lymphoma) | No | 4,003 | 8,802.7 | 37 (0.92) | 0.42 | - | 0.179 | - | |  |
|  | Yes | 533 | 1,256.2 | 5 (0.94) | 0.40 | 0.95 (0.37-2.42) |  | - | |  |
|  | Unknown | 2,485 | 5,393.8 | 35 (1.41) | 0.65 | 1.52 (0.96-2.41) |  | - | |  |

Multivariate analysis was performed for all factors with *p*<0.1 in the univariate analysis, with *p*<0.05 indicating statistical significance.

AE, adverse event; CI, confidence interval; CV, cardiovascular; HDL, high-density lipoprotein; HR, hazard ratio; HZ, herpes zoster; IR, incidence rate; MACE, major adverse cardiovascular events; ND, not detected; PY, patient-years; RA, rheumatoid arthritis.

**TABLE S4** Patients’ background factors for the incidence of malignancies

| **Background factors** | | **Number of patients in the safety analysis set (N)** | **PY** | **Number of patients with AEs**  **n (%)** | **IR/100 PY** | **Univariate analysis** | | **Multivariate analysis** | | |
| --- | --- | --- | --- | --- | --- | --- | --- | --- | --- | --- |
|  |  |  |  |  |  | **HR (95% CI)** | ***p*-value** | **HR (95% CI)** | ***p*-value** |  |
| Smoking history | No | 4,833 | 10,632.0 | 157 (3.25) | 1.48 | - | 0.004 | - | 0.007 |  |
|  | Yes | 662 | 1,427.1 | 36 (5.44) | 2.52 | 1.71 (1.19-2.45) |  | 1.65 (1.11-2.46) |  |  |
|  | Unknown | 1,526 | 3,212.7 | 40 (2.62) | 1.25 | 0.84 (0.59-1.19) |  | 0.77 (0.54-1.10) |  |  |
| Concomitant hypertension | No | 5,325 | 11,510.0 | 162 (3.04) | 1.41 | - | <0.001 | - |  |  |
|  | Yes | 1,695 | 3,759.0 | 70 (4.13) | 1.86 | 1.33 (1.01-1.76) |  | - |  |  |
|  | Unknown | 1 | 2.8 | 1 (100.00) | 35.32 | 25.42 (3.56-181.61) |  | - |  |  |
| HDL <40 mg/dL during treatment | No | 1,274 | 2,826.1 | 41 (3.22) | 1.45 | - | 0.559 | - |  |  |
|  | Yes | 130 | 256.6 | 6 (4.62) | 2.34 | 1.60 (0.68-3.77) |  | - |  |  |
|  | Unknown | 5,617 | 12,189.0 | 186 (3.31) | 1.53 | 1.05 (0.75-1.47) |  | - |  |  |
| Concomitant diabetes mellitus | No | 6,227 | 13,558.8 | 185 (2.97) | 1.36 | - | <0.001 | - | 0.003 |  |
|  | Yes | 793 | 1,710.2 | 47 (5.93) | 2.75 | 2.02 (1.47-2.78) |  | 1.55 (1.12-2.16) |  |  |
|  | Unknown | 1 | 2.8 | 1 (100.00) | 35.32 | 26.18 (3.67-186.89) |  | 0.04 (<0.01 to 0.75) |  |  |
| History of coronary artery disease | No | 6,922 | 15,064.6 | 227 (3.28) | 1.51 | - | 0.004 | - |  |  |
|  | Yes | 98 | 204.4 | 5 (5.10) | 2.45 | 1.65 (0.68-4.01) |  | - |  |  |
|  | Unknown | 1 | 2.8 | 1 (100.00) | 35.32 | 23.71 (3.32-169.08) |  | - |  |  |
| Presence of extra-articular disease associated with RA | No | 6,748 | 14,682.5 | 217 (3.22) | 1.48 | - | 0.001 | - |  |  |
|  | Yes | 272 | 586.5 | 15 (5.51) | 2.56 | 1.69 (1.00-2.86) |  | - |  |  |
|  | Unknown | 1 | 2.8 | 1 (100.00) | 35.32 | 24.12 (3.38-172.08) |  | - |  |  |
| Sex | Male | 1,423 | 2,993.0 | 78 (5.48) | 2.61 | - | <0.001 | - | 0.004 |  |
|  | Female | 5,598 | 12,278.8 | 155 (2.77) | 1.26 | 0.48 (0.37-0.63) |  | 0.63 (0.46-0.86) |  |  |
| Age (years) | <50 | 1,112 | 2,493.5 | 17 (1.53) | 0.68 | - | <0.001 | - | <0.001 |  |
|  | ≥50 to <65 | 2,284 | 5,250.2 | 57 (2.50) | 1.09 | 1.59 (0.92-2.73) |  | 1.60 (0.91-2.79) |  |  |
|  | ≥65 | 3,625 | 7,528.1 | 159 (4.39) | 2.11 | 3.11 (1.88-5.12) |  | 3.12 (1.85-5.28) |  |  |
| Weight (kg) | <50 | 1,955 | 4,259.0 | 44 (2.25) | 1.03 | - | 0.011 | - | 0.020 |  |
|  | ≥50 to <60 | 1,979 | 4,439.1 | 88 (4.45) | 1.98 | 1.93 (1.34-2.77) |  | 1.83 (1.27-2.65) |  |  |
|  | ≥60 to <70 | 1,084 | 2,350.9 | 38 (3.51) | 1.62 | 1.57 (1.02-2.43) |  | 1.35 (0.86-2.13) |  |  |
|  | ≥70 | 594 | 1,321.0 | 19 (3.20) | 1.44 | 1.39 (0.81-2.38) |  | 1.25 (0.70-2.24) |  |  |
|  | Unknown | 1,409 | 2,901.8 | 44 (3.12) | 1.52 | 1.45 (0.96-2.20) |  | 1.61 (1.04-2.48) |  |  |
| Stage of RA | I-II | 3,109 | 6,586.5 | 105 (3.38) | 1.59 | - | 0.029 | - |  |  |
|  | III | 1,767 | 3,888.1 | 68 (3.85) | 1.75 | 1.10 (0.81-1.50) |  | - |  |  |
|  | IV | 2,106 | 4,743.5 | 57 (2.71) | 1.20 | 0.76 (0.55-1.05) |  | - |  |  |
|  | Unknown | 39 | 53.6 | 3 (7.69) | 5.60 | 3.26 (1.03-10.30) |  | - |  |  |
| Class of RA | 1-2 | 5,276 | 11,525.4 | 158 (2.99) | 1.37 | - | 0.014 | - |  |  |
|  | 3 | 1,544 | 3,374.8 | 64 (4.15) | 1.90 | 1.37 (1.03-1.83) |  | - |  |  |
|  | 4 | 166 | 320.2 | 8 (4.82) | 2.50 | 1.83 (0.90-3.72) |  | - |  |  |
|  | Unknown | 35 | 51.4 | 3 (8.57) | 5.84 | 3.68 (1.16-11.60) |  | - |  |  |
| Duration of disease (years) | <2 | 787 | 1,548.5 | 24 (3.05) | 1.55 | - | 0.973 | - |  |  |
|  | ≥2 to <5 | 1,052 | 2,281.5 | 36 (3.42) | 1.58 | 0.99 (0.59-1.66) |  | - |  |  |
|  | ≥5 to <10 | 1,394 | 3,048.7 | 47 (3.37) | 1.54 | 0.98 (0.60-1.60) |  | - |  |  |
|  | ≥10 to <20 | 1,841 | 4,210.9 | 60 (3.26) | 1.42 | 0.90 (0.56-1.45) |  | - |  |  |
|  | ≥20 | 1,133 | 2,537.7 | 37 (3.27) | 1.46 | 0.92 (0.55-1.54) |  | - |  |  |
|  | Unknown | 814 | 1,644.5 | 29 (3.56) | 1.76 | 1.09 (0.63-1.88) |  | - |  |  |
| History of infection | No | 6,061 | 13,126.2 | 184 (3.04) | 1.40 | - | <0.001 | - | 0.014 |  |
|  | Yes | 959 | 2,142.7 | 48 (5.01) | 2.24 | 1.62 (1.18-2.22) |  | 1.50 (1.09-2.07) |  |  |
|  | Unknown | 1 | 2.8 | 1 (100.00) | 35.32 | 25.55 (3.58-182.40) |  | 0.00 (ND) |  |  |
| History of HZ | No | 6,659 | 14,417.2 | 217 (3.26) | 1.51 | - | 0.006 | - |  |  |
|  | Yes | 361 | 851.7 | 15 (4.16) | 1.76 | 1.18 (0.70-1.99) |  | - |  |  |
|  | Unknown | 1 | 2.8 | 1 (100.00) | 35.32 | 23.74 (3.33-169.32) |  | - |  |  |
| History of malignancies | No | 6,737 | 14,688.9 | 215 (3.19) | 1.46 | - | <0.001 | - |  |  |
|  | Yes | 283 | 580.1 | 17 (6.01) | 2.93 | 2.04 (1.25-3.35) |  | - |  |  |
|  | Unknown | 1 | 2.8 | 1 (100.00) | 35.32 | 24.44 (3.43-174.34) |  | - |  |  |
| History of CV disease | No | 6,628 | 14,444.1 | 216 (3.26) | 1.50 | - | 0.004 | - |  |  |
|  | Yes | 392 | 824.8 | 16 (4.08) | 1.94 | 1.29 (0.78-2.15) |  | - |  |  |
|  | Unknown | 1 | 2.8 | 1 (100.00) | 35.32 | 23.87 (3.35-170.30) |  | - |  |  |
| History of lung disorder | No | 6,694 | 14,554.0 | 213 (3.18) | 1.46 | - | <0.001 | - |  |  |
|  | Yes | 326 | 714.9 | 19 (5.83) | 2.66 | 1.78 (1.11-2.84) |  | - |  |  |
|  | Unknown | 1 | 2.8 | 1 (100.00) | 35.32 | 24.35 (3.41-173.73) |  | - |  |  |
| History of interstitial pneumonia | No | 6,785 | 14,754.1 | 220 (3.24) | 1.49 | - | 0.003 | - |  |  |
|  | Yes | 235 | 514.9 | 12 (5.11) | 2.33 | 1.52 (0.85-2.72) |  | - |  |  |
|  | Unknown | 1 | 2.8 | 1 (100.00) | 35.32 | 23.91 (3.35-170.55) |  | - |  |  |
| History of metabolic abnormality | No | 6,923 | 15,034.4 | 226 (3.26) | 1.50 | - | <0.001 | - | <0.001 |  |
|  | Yes | 96 | 234.4 | 5 (5.21) | 2.13 | 1.38 (0.57-3.35) |  | 1.24 (0.51-3.03) |  |  |
|  | Unknown | 2 | 2.9 | 2 (100.00) | 67.83 | 45.14  (11.23-181.47) |  | 673.21  (80.94-ND) |  |  |
| Complication (infection) | No | 6,726 | 14,606.2 | 216 (3.21) | 1.48 | - | 0.001 | - |  |  |
|  | Yes | 294 | 662.7 | 16 (5.44) | 2.41 | 1.63 (0.98-2.71) |  | - |  |  |
|  | Unknown | 1 | 2.8 | 1 (100.00) | 35.32 | 24.14 (3.38-172.24) |  | - |  |  |
| Complication (HZ) | No | 7,015 | 15,256.6 | 232 (3.31) | 1.52 | - | 0.007 | - |  |  |
|  | Yes | 5 | 12.3 | 0 (0.00) | 0.00 | 0.00 (ND) |  | - |  |  |
|  | Unknown | 1 | 2.8 | 1 (100.00) | 35.32 | 23.48 (3.29-167.45) |  | - |  |  |
| Complication (malignancies) | No | 6,980 | 15,221.1 | 213 (3.05) | 1.40 | - | <0.001 | - | <0.001 |  |
|  | Yes | 40 | 47.8 | 19 (47.50) | 39.75 | 29.17 (18.17-46.82) |  | 28.23 (17.30-46.06) |  |  |
|  | Unknown | 1 | 2.8 | 1 (100.00) | 35.32 | 25.53 (3.58-182.02) |  | 0.00 (ND) |  |  |
| Complication (CV disease) | No | 5,103 | 11,053.7 | 152 (2.98) | 1.38 | - | <0.001 | - |  |  |
|  | Yes | 1,917 | 4,215.2 | 80 (4.17) | 1.90 | 1.39 (1.06-1.82) |  | - |  |  |
|  | Unknown | 1 | 2.8 | 1 (100.00) | 35.32 | 26.01 (3.64-185.89) |  | - |  |  |
| Complication (lung disorder) | No | 6,288 | 13,732.3 | 194 (3.09) | 1.41 | - | <0.001 | - |  |  |
|  | Yes | 732 | 1,536.7 | 38 (5.19) | 2.47 | 1.76 (1.24-2.49) |  | - |  |  |
|  | Unknown | 1 | 2.8 | 1 (100.00) | 35.32 | 25.29 (3.54-180.48) |  | - |  |  |
| Complication (interstitial pneumonia) | No | 6,563 | 14,340.5 | 209 (3.18) | 1.46 | - | <0.001 | - |  |  |
|  | Yes | 457 | 928.4 | 23 (5.03) | 2.48 | 1.71 (1.11-2.63) |  | - |  |  |
|  | Unknown | 1 | 2.8 | 1 (100.00) | 35.32 | 24.51 (3.44-174.90) |  | - |  |  |
| Complication (metabolic abnormality) | No | 5,097 | 11,025.4 | 150 (2.94) | 1.36 | - | <0.001 | - |  |  |
|  | Yes | 1,922 | 4,243.4 | 81 (4.21) | 1.91 | 1.41 (1.07-1.84) |  | - |  |  |
|  | Unknown | 2 | 2.9 | 2 (100.00) | 67.83 | 49.92  (12.38-201.35) |  | - |  |  |
| Status of methotrexate use (dose at baseline; mg/week) | No | 2,846 | 5,936.1 | 98 (3.44) | 1.65 | - | 0.754 | - |  |  |
|  | ≤8 | 2,116 | 4,637.3 | 65 (3.07) | 1.40 | 0.85 (0.62-1.16) |  | - |  |  |
|  | >8 | 2,058 | 4,695.4 | 70 (3.40) | 1.49 | 0.89 (0.65-1.21) |  | - |  |  |
|  | Unknown | 1 | 3.0 | 0 (0.00) | 0.00 | 0.00 (ND) |  | - |  |  |
| Status of oral steroid use (dose at baseline; mg/day) | No | 3,159 | 6,886.3 | 105 (3.32) | 1.52 | - | 0.672 | - |  |  |
|  | <2.5 | 991 | 2,162.2 | 33 (3.33) | 1.53 | 1.01 (0.68-1.50) |  | - |  |  |
|  | ≥2.5 to <5 | 1,815 | 3,945.9 | 56 (3.09) | 1.42 | 0.94 (0.68-1.30) |  | - |  |  |
|  | ≥5 | 957 | 2,060.6 | 33 (3.45) | 1.60 | 1.05 (0.71-1.56) |  | - |  |  |
|  | Unknown | 99 | 216.7 | 6 (6.06) | 2.77 | 1.80 (0.79-4.11) |  | - |  |  |
| History of tacrolimus use | No | 5,957 | 12,895.6 | 193 (3.24) | 1.50 | - | 0.836 | - |  |  |
|  | Yes | 967 | 2,186.5 | 37 (3.83) | 1.69 | 1.11 (0.78-1.58) |  | - |  |  |
|  | Unknown | 97 | 189.6 | 3 (3.09) | 1.58 | 1.08 (0.34-3.36) |  | - |  |  |
| History of biological agent use | No | 3,329 | 7,250.7 | 88 (2.64) | 1.21 | - | 0.003 | - | 0.003 |  |
|  | Yes | 3,692 | 8,021.1 | 145 (3.93) | 1.81 | 1.50 (1.15-1.96) |  | 1.50 (1.15-1.96) |  |  |
|  | Unknown | 0 | - | - | - | 0.00 (ND) |  | 0.00 (ND) |  |  |
| Initial tofacitinib dose (mg/day) | 5 | 2,343 | 4,957.7 | 69 (2.94) | 1.39 | - | 0.643 | - |  |  |
|  | 10 | 4,640 | 10,228.4 | 164 (3.53) | 1.60 | 1.14 (0.86-1.52) |  | - |  |  |
|  | Unknown | 38 | 85.7 | 0 (0.00) | 0.00 | 0.00 (ND) |  | - |  |  |
| Family history of malignancies (including lymphoma) | No | 4,003 | 8,715.3 | 120 (3.00) | 1.38 | - | 0.253 | - |  |  |
|  | Yes | 533 | 1,237.5 | 22 (4.13) | 1.78 | 1.29 (0.82-2.03) |  | - |  |  |
|  | Unknown | 2,485 | 5,319.0 | 91 (3.66) | 1.71 | 1.23 (0.94-1.62) |  | - |  |  |

Multivariate analysis was performed for all factors with *p*<0.1 in the univariate analysis, with *p*<0.05 indicating statistical significance.

AE, adverse event; CI, confidence interval; CV, cardiovascular; HDL, high-density lipoprotein; HR, hazard ratio; HZ, herpes zoster; IR, incidence rate; ND, not detected; PY, patient-years; RA, rheumatoid arthritis.

**TABLE S5** Patients’ background factors for the incidence of SI

| **Background factors** | | **Number of patients in the safety analysis set (N)** | **PY** | **Number of patients with AEs**  **n (%)** | **IR/100 PY** | **Univariate analysis** | | **Multivariate analysis** | |
| --- | --- | --- | --- | --- | --- | --- | --- | --- | --- |
|  |  |  |  |  |  | **HR (95% CI)** | ***p*-value** | **HR (95% CI)** | ***p*-value** |
| Smoking history | No | 4,833 | 10,193.8 | 420 (8.69) | 4.12 | - | 0.309 | - |  |
|  | Yes | 662 | 1,395.5 | 59 (8.91) | 4.23 | 1.02 (0.78-1.35) |  | - |  |
|  | Unknown | 1,526 | 3,095.0 | 110 (7.21) | 3.55 | 0.85 (0.69-1.05) |  | - |  |
| Concomitant hypertension | No | 5,325 | 11,151.6 | 389 (7.31) | 3.49 | - | <0.001 | - |  |
|  | Yes | 1,695 | 3,529.6 | 200 (11.80) | 5.67 | 1.61 (1.36-1.91) |  | - |  |
|  | Unknown | 1 | 3.0 | 0 (0.00) | 0.00 | 0.00 (ND) |  | - |  |
| HDL <40 mg/dL during treatment | No | 1,274 | 2,705.5 | 109 (8.56) | 4.03 | - | 0.475 | - |  |
|  | Yes | 130 | 245.5 | 14 (10.77) | 5.70 | 1.37 (0.78-2.38) |  | - |  |
|  | Unknown | 5,617 | 11,733.3 | 466 (8.30) | 3.97 | 0.98 (0.80-1.21) |  | - |  |
| Concomitant diabetes mellitus | No | 6,227 | 13,084.4 | 475 (7.63) | 3.63 | - | <0.001 | - | 0.004 |
|  | Yes | 793 | 1,596.9 | 114 (14.38) | 7.14 | 1.95 (1.59-2.39) |  | 1.44 (1.16-1.78) |  |
|  | Unknown | 1 | 3.0 | 0 (0.00) | 0.00 | 0.00 (ND) |  | 0.00 (ND) |  |
| History of coronary artery disease | No | 6,922 | 14,500.7 | 570 (8.23) | 3.93 | - | <0.001 | - | 0.036 |
|  | Yes | 98 | 180.5 | 19 (19.39) | 10.53 | 2.56 (1.62-4.05) |  | 1.65 (1.03-2.63) |  |
|  | Unknown | 1 | 3.0 | 0 (0.00) | 0.00 | 0.00 (ND) |  | 0.00 (ND) |  |
| Presence of extra-articular disease associated with RA | No | 6,748 | 14,134.2 | 549 (8.14) | 3.88 | - | <0.001 | - |  |
|  | Yes | 272 | 547.1 | 40 (14.71) | 7.31 | 1.87 (1.35-2.57) |  | - |  |
|  | Unknown | 1 | 3.0 | 0 (0.00) | 0.00 | 0.00 (ND) |  | - |  |
| Sex | Male | 1,423 | 2,880.1 | 139 (9.77) | 4.83 | - | 0.017 | - |  |
|  | Female | 5,598 | 11,804.1 | 450 (8.04) | 3.81 | 0.79 (0.66-0.96) |  | - |  |
| Age (years) | <50 | 1,112 | 2,451.1 | 39 (3.51) | 1.59 | - | <0.001 | - | <0.001 |
|  | ≥50 to <65 | 2,284 | 5,060.5 | 161 (7.05) | 3.18 | 2.00 (1.41-2.84) |  | 1.85 (1.30-2.63) |  |
|  | ≥65 | 3,625 | 7,172.6 | 389 (10.73) | 5.42 | 3.31 (2.38-4.60) |  | 2.71 (1.93-3.80) |  |
| Weight (kg) | <50 | 1,955 | 4,016.5 | 210 (10.74) | 5.23 | - | <0.001 | - | 0.002 |
|  | ≥50 to <60 | 1,979 | 4,309.5 | 149 (7.53) | 3.46 | 0.67 (0.54-0.83) |  | 0.72 (0.58-0.89) |  |
|  | ≥60 to <70 | 1,084 | 2,272.9 | 92 (8.49) | 4.05 | 0.78 (0.61-0.99) |  | 0.83 (0.65-1.07) |  |
|  | ≥70 | 594 | 1,245.8 | 56 (9.43) | 4.50 | 0.86 (0.64-1.16) |  | 1.04 (0.76-1.41) |  |
|  | Unknown | 1,409 | 2,839.6 | 82 (5.82) | 2.89 | 0.55 (0.43-0.71) |  | 0.65 (0.50-0.85) |  |
| Stage of RA | I-II | 3,109 | 6,407.0 | 204 (6.56) | 3.18 | - | <0.001 | - |  |
|  | III | 1,767 | 3,777.8 | 145 (8.21) | 3.84 | 1.21 (0.98-1.50) |  | - |  |
|  | IV | 2,106 | 4,449.0 | 233 (11.06) | 5.24 | 1.65 (1.37-2.00) |  | - |  |
|  | Unknown | 39 | 50.4 | 7 (17.95) | 13.89 | 3.99 (1.88-8.48) |  | - |  |
| Class of RA | 1-2 | 5,276 | 11,135.0 | 383 (7.26) | 3.44 | - | <0.001 | - | <0.001 |
|  | 3 | 1,544 | 3,195.6 | 177 (11.46) | 5.54 | 1.60 (1.34-1.91) |  | 1.21 (1.00-1.45) |  |
|  | 4 | 166 | 305.4 | 22 (13.25) | 7.20 | 2.02 (1.31-3.11) |  | 1.20 (0.77-1.85) |  |
|  | Unknown | 35 | 48.2 | 7 (20.00) | 14.52 | 3.82 (1.81-8.07) |  | 4.58 (2.12-9.86) |  |
| Duration of disease (years) | <2 | 787 | 1,524.0 | 43 (5.46) | 2.82 | - | <0.001 | - | 0.010 |
|  | ≥2 to <5 | 1,052 | 2,233.0 | 67 (6.37) | 3.00 | 1.08 (0.74-1.58) |  | 1.03 (0.70-1.51) |  |
|  | ≥5 to <10 | 1,394 | 2,945.9 | 110 (7.89) | 3.73 | 1.35 (0.95-1.92) |  | 1.31 (0.92-1.87) |  |
|  | ≥10 to <20 | 1,841 | 4,018.5 | 183 (9.94) | 4.55 | 1.65 (1.19-2.31) |  | 1.54 (1.10-2.16) |  |
|  | ≥20 | 1,133 | 2,375.9 | 127 (11.21) | 5.35 | 1.92 (1.36-2.71) |  | 1.61 (1.13-2.30) |  |
|  | Unknown | 814 | 1,587.1 | 59 (7.25) | 3.72 | 1.31 (0.88-1.94) |  | 1.28 (0.86-1.92) |  |
| History of infection | No | 6,061 | 12,706.5 | 435 (7.18) | 3.42 | - | <0.001 | - | <0.001 |
|  | Yes | 959 | 1,974.8 | 154 (16.06) | 7.80 | 2.27 (1.89-2.73) |  | 1.72 (1.42-2.07) |  |
|  | Unknown | 1 | 3.0 | 0 (0.00) | 0.00 | 0.00 (ND) |  | 0.00 (ND) |  |
| History of HZ | No | 6,659 | 13,867.5 | 546 (8.20) | 3.94 | - | 0.146 | - |  |
|  | Yes | 361 | 813.7 | 43 (11.91) | 5.28 | 1.36 (1.00-1.86) |  | - |  |
|  | Unknown | 1 | 3.0 | 0 (0.00) | 0.00 | 0.00 (ND) |  | - |  |
| History of malignancies | No | 6,737 | 14,117.1 | 554 (8.22) | 3.92 | - | 0.035 | - |  |
|  | Yes | 283 | 564.1 | 35 (12.37) | 6.20 | 1.57 (1.12-2.21) |  | - |  |
|  | Unknown | 1 | 3.0 | 0 (0.00) | 0.00 | 0.00 (ND) |  | - |  |
| History of CV disease | No | 6,628 | 13,913.5 | 532 (8.03) | 3.82 | - | <0.001 | - |  |
|  | Yes | 392 | 767.8 | 57 (14.54) | 7.42 | 1.89 (1.44-2.49) |  | - |  |
|  | Unknown | 1 | 3.0 | 0 (0.00) | 0.00 | 0.00 (ND) |  | - |  |
| History of lung disorder | No | 6,694 | 14,023.1 | 538 (8.04) | 3.84 | - | <0.001 | - | 0.006 |
|  | Yes | 326 | 658.1 | 51 (15.64) | 7.75 | 2.01 (1.51-2.67) |  | 1.51 (1.12-2.02) |  |
|  | Unknown | 1 | 3.0 | 0 (0.00) | 0.00 | 0.00 (ND) |  | 0.00 (ND) |  |
| History of interstitial pneumonia | No | 6,785 | 14,202.8 | 552 (8.14) | 3.89 | - | <0.001 | - |  |
|  | Yes | 235 | 478.4 | 37 (15.74) | 7.73 | 1.98 (1.42-2.77) |  | - |  |
|  | Unknown | 1 | 3.0 | 0 (0.00) | 0.00 | 0.00 (ND) |  | - |  |
| History of metabolic abnormality | No | 6,923 | 14,452.3 | 580 (8.38) | 4.01 | - | 0.999 | - |  |
|  | Yes | 96 | 225.9 | 9 (9.38) | 3.98 | 1.00 (0.52-1.94) |  | - |  |
|  | Unknown | 2 | 6.0 | 0 (0.00) | 0.00 | 0.00 (ND) |  | - |  |
| Complication (infection) | No | 6,726 | 14,079.5 | 545 (8.10) | 3.87 | - | <0.001 | - |  |
|  | Yes | 294 | 601.7 | 44 (14.97) | 7.31 | 1.88 (1.38-2.55) |  | - |  |
|  | Unknown | 1 | 3.0 | 0 (0.00) | 0.00 | 0.00 (ND) |  | - |  |
| Complication (HZ) | No | 7,015 | 14,668.9 | 589 (8.40) | 4.02 | - | 0.999 | - |  |
|  | Yes | 5 | 12.3 | 0 (0.00) | 0.00 | 0.00 (ND) |  | - |  |
|  | Unknown | 1 | 3.0 | 0 (0.00) | 0.00 | 0.00 (ND) |  | - |  |
| Complication (malignancies) | No | 6,980 | 14,625.6 | 582 (8.34) | 3.98 | - | 0.029 | - | 0.035 |
|  | Yes | 40 | 55.6 | 7 (17.50) | 12.58 | 2.76 (1.31-5.81) |  | 2.25 (1.06-4.78) |  |
|  | Unknown | 1 | 3.0 | 0 (0.00) | 0.00 | 0.00 (ND) |  | 0.00 (ND) |  |
| Complication (CV disease) | No | 5,103 | 10,716.6 | 363 (7.11) | 3.39 | - | <0.001 | - |  |
|  | Yes | 1,917 | 3,964.6 | 226 (11.79) | 5.70 | 1.67 (1.41-1.97) |  | - |  |
|  | Unknown | 1 | 3.0 | 0 (0.00) | 0.00 | 0.00 (ND) |  | - |  |
| Complication (lung disorder) | No | 6,288 | 13,294.3 | 449 (7.14) | 3.38 | - | <0.001 | - | <0.001 |
|  | Yes | 732 | 1,387.0 | 140 (19.13) | 10.09 | 2.91 (2.41-3.52) |  | 2.68 (2.04-3.50) |  |
|  | Unknown | 1 | 3.0 | 0 (0.00) | 0.00 | 0.00 (ND) |  | 0.00 (ND) |  |
| Complication (interstitial pneumonia) | No | 6,563 | 13,826.2 | 511 (7.79) | 3.70 | - | <0.001 | - | 0.042 |
|  | Yes | 457 | 855.0 | 78 (17.07) | 9.12 | 2.39 (1.89-3.04) |  | 0.70 (0.50-0.99) |  |
|  | Unknown | 1 | 3.0 | 0 (0.00) | 0.00 | 0.00 (ND) |  | 0.00 (ND) |  |
| Complication (metabolic abnormality) | No | 5,097 | 10,681.3 | 361 (7.08) | 3.38 | - | <0.001 | - |  |
|  | Yes | 1,922 | 3,996.9 | 228 (11.86) | 5.70 | 1.68 (1.42-1.98) |  | - |  |
|  | Unknown | 2 | 6.0 | 0 (0.00) | 0.00 | 0.00 (ND) |  | - |  |
| Status of methotrexate use (dose at baseline; mg/week) | No | 2,846 | 5,708.9 | 262 (9.21) | 4.59 | - | 0.025 | - |  |
|  | ≤8 | 2,116 | 4,434.8 | 178 (8.41) | 4.01 | 0.88 (0.73-1.07) |  | - |  |
|  | >8 | 2,058 | 4,537.5 | 149 (7.24) | 3.28 | 0.73 (0.60-0.89) |  | - |  |
|  | Unknown | 1 | 3.0 | 0 (0.00) | 0.00 | 0.00 (ND) |  | - |  |
| Status of oral steroid use (dose at baseline; mg/day) | No | 3,159 | 6,698.6 | 212 (6.71) | 3.16 | - | <0.001 | - | <0.001 |
|  | <2.5 | 991 | 2,087.4 | 71 (7.16) | 3.40 | 1.07 (0.82-1.41) |  | 0.95 (0.73-1.25) |  |
|  | ≥2.5 to <5 | 1,815 | 3,775.4 | 160 (8.82) | 4.24 | 1.33 (1.09-1.64) |  | 1.20 (0.98-1.48) |  |
|  | ≥5 | 957 | 1,906.7 | 143 (14.94) | 7.50 | 2.35 (1.90-2.90) |  | 2.07 (1.66-2.58) |  |
|  | Unknown | 99 | 216.1 | 3 (3.03) | 1.39 | 0.44 (0.14-1.38) |  | 0.42 (0.13-1.30) |  |
| History of tacrolimus use | No | 5,957 | 12,403.8 | 495 (8.31) | 3.99 | - | 0.528 | - |  |
|  | Yes | 967 | 2,095.5 | 89 (9.20) | 4.25 | 1.08 (0.86-1.35) |  | - |  |
|  | Unknown | 97 | 184.9 | 5 (5.15) | 2.70 | 0.67 (0.28-1.61) |  | - |  |
| History of biological agent use | No | 3,329 | 6,986.4 | 262 (7.87) | 3.75 | - | 0.149 | - |  |
|  | Yes | 3,692 | 7,697.8 | 327 (8.86) | 4.25 | 1.13 (0.96-1.33) |  | - |  |
|  | Unknown | 0 | - | - | - | 0.00 (ND) |  | - |  |
| Initial tofacitinib dose (mg/day) | 5 | 2,343 | 4,782.8 | 192 (8.19) | 4.01 | - | 0.981 | - |  |
|  | 10 | 4,640 | 9,816.7 | 394 (8.49) | 4.01 | 1.01 (0.85-1.20) |  | - |  |
|  | Unknown | 38 | 84.7 | 3 (7.89) | 3.54 | 0.92 (0.29-2.87) |  | - |  |
| Family history of malignancies (including lymphoma) | No | 4,003 | 8,381.9 | 320 (7.99) | 3.82 | - | 0.184 | - |  |
|  | Yes | 533 | 1,189.3 | 42 (7.88) | 3.53 | 0.94 (0.68-1.30) |  | - |  |
|  | Unknown | 2,485 | 5,113.0 | 227 (9.13) | 4.44 | 1.16 (0.98-1.37) |  | - |  |

Multivariate analysis was performed for all factors with *p*<0.1 in the univariate analysis, with *p*<0.05 indicating statistical significance.

AE, adverse event; CI, confidence interval; CV, cardiovascular; HDL, high-density lipoprotein; HR, hazard ratio; HZ, herpes zoster; IR, incidence rate; ND, not detected; PY, patient-years; RA, rheumatoid arthritis; SI, serious infection.
